# Red-light-excited dynamic near-infrared organic afterglow materials for in vivo bioimaging

**DOI:** 10.1038/s41377-026-02340-3

**Published:** 2026-06-10

**Authors:** Lei Zhou, Jiacheng Yang, Zhenyi He, Zhiqin Wu, Ping Jiang, Jinming Song, Liangwei Ma, He Tian, Xiang Ma

**Affiliations:** Key Laboratory for Advanced Materials and Feringa Nobel Prize Scientist Joint Research Center, Frontiers Science Center for Materiobiology and Dynamic Chemistry, Institute of Fine Chemicals, School of Chemistry and Molecular Engineering, East China University of Science and Technology, Meilong Road 130, Shanghai, 200237 China

**Keywords:** Optical materials and structures, Optical physics

## Abstract

Extending the excitation and emission wavelengths into the red or even near-infrared region is a highly challenging yet scientifically valuable research topic in the field of organic afterglow materials. To solve this issue, we put forward a twisted intramolecular charge transfer dopant molecule design strategy, in which long-lived electron-deficient dopant is decorated with electron-rich substituent. In this way, the orbital energy level of the dopant can be lowered while maintaining the compatibility with the host. Benefiting from the twisted molecular conformation and small energy gap, the obtained dopant (**CN**) shows visible-light-excited afterglow with various performance (decay path, lifetime, emission wavelength) when doped into different matrices. Particularly, the maximum excitation wavelength extends to 567 nm and tails to 700 nm when **CN** is doped into benzophenone matrix. More importantly, the maximum emission wavelength of the afterglow extends to 725 nm (τ = 67.82 ms). We also successfully apply this material in autofluorescence-free bioimaging. This work provides a viable molecular design strategy for developing red-light-excitable near-infrared afterglow materials and demonstrates their potential for in vivo bioimaging.

## Abstract (web summary)

This work develops red-light-excitable dynamic near-infrared afterglow materials and demonstrates their potential for in vivo bioimaging.

## Graphical abstract

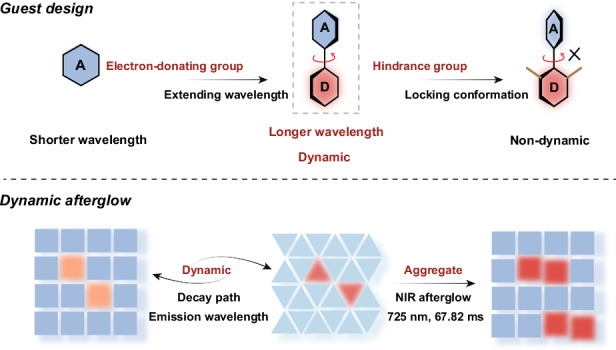

## Introduction

Organic afterglow materials have received widespread attention due to their characteristic long emission lifetimes^1–7^. The prolonged emission lifetime of these materials adds a time dimension to traditional optical properties such as emission wavelength, intensity, or polarization^8–11^. This makes them uniquely advantageous for applications in bioimaging, advanced anti-counterfeiting, information encryption, sensing, and other fields compared to traditional fluorescent materials^12–16^. Although many high-performance organic afterglow materials have been successfully developed in recent years, most of the existing material systems face the issue of short excitation wavelengths (<450 nm), mainly in the ultraviolet region^17–23^. In contrast, visible light, especially red light, offers significant advantages in reducing phototoxicity, enhancing photostability, and improving penetration depth^24–27^. Developing visible-light-excited organic afterglow materials is crucial for expanding their practical applications. Particularly in the field of autofluorescence-free bioimaging and biosensing, the ability to be excited by long wavelength light and emission in the near-infrared region are essential requirements. Molecules designed for visible light excitation (absorption) materials often require extending the conjugation of dyes or constructing donor-acceptor (D-A) molecular systems^28–32^. However, the frontier molecular orbitals of such compounds are typically π-type, making it difficult to achieve sufficient spin-orbit coupling. To promote the utilization of triplet excitons, heavy atoms like bromine and iodine are often introduced into the molecular framework^19,33–38^. While these heavy atoms enhance the intersystem crossing (ISC) rate, they also increase the radiative transition rate of the lowest triple state (T_1_), thereby significantly reducing phosphorescence lifetime. The bi-component doping strategy can bypass the ISC process of the dopant molecules by utilizing interactions between the host and dopant to directly generate the triplet excited state of guest, thereby achieving long-lived afterglow^39–41^. Therefore, this strategy holds unique advantages in developing long-wavelength-excited afterglow materials. However, matching the host and dopant in bi-component doping materials is often based on semi-empirical trial-and-error methods, lacking effective theoretical guidance, which greatly limits the development of novel organic afterglow materials^42,43^.

Recently, a series of red afterglow materials based on pyrene was developed^24^. Notably, even after modifying with strong electron-donating structures such as methoxybenzene or *N, N*-dimethylaniline, the obtained derivatives still interact with the matrix to produce afterglow. These results hint that the grafting of electron-donating group does not change the compatibility with the host. This insight inspired us to utilize existing electron-deficient dopants as parent compounds and modify them with electron-rich substituents, such as *N, N*-dimethylaniline, to construct twisted intramolecular charge transfer (TICT)-type dopants. And if structural modifications maintain the compatibility with the host, this could significantly broaden the excitation and emission wavelengths of the resulting materials, enabling visible-light-excited, or even red-light-excited afterglow materials.

**Fig. 1:**
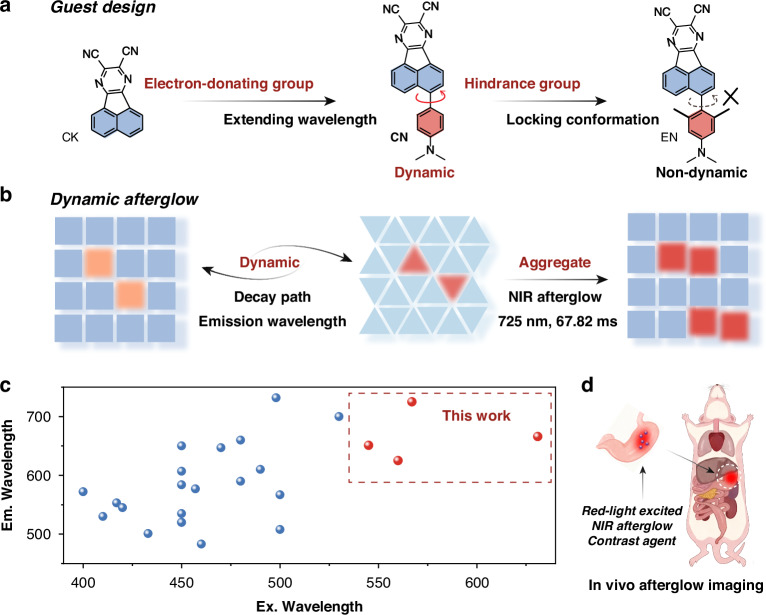
Schematic illustration of constructing red-light-excited near-infrared (NIR) dynamic afterglow materials and in vivo afterglow bioimaging. **a** Design principles for guest molecules; **b** Schematic diagram of dynamic and near-infrared afterglow; **c** Comparison of emission and excitation wavelength of this work with other previous work based on visible-light-excited afterglow materials (Table S1); **d** Schematic diagram of in vivo afterglow bioimaging

In the preliminary studies, we found electron-deficient compound **CK** could exhibit decent yellow afterglow after doping into benzophenone (**BPO**) matrix with an 87.25 ms lifetime. To verify our hypothesis, we grafted *N, N*-dimethylaniline into **CK** skeleton to construct a TICT-type dopant (**CN**, Fig. 1a). The results showed that, compound **CN** exhibited a clear afterglow centered at 725 nm with a 67.82 ms lifetime when doped into **BPO** matrix. Importantly, the maximum excitation wavelength extends to 567 nm and tails to 700 nm. Benefiting from flexibility of the molecular skeleton, the modulation of the afterglow emission decay path, lifetime, and wavelength could be achieved by changing the matrices. By adjusting the host materials, the afterglow emission wavelength could be tuned from 625 nm to 725 nm (Fig. 1b, c). We also successfully applied this material in autofluorescence-free bioimaging Fig. 1d. This work provides a viable molecular design strategy for developing red-light-excitable near-infrared afterglow materials suitable for in vivo bioimaging.

## Results

### The photophysical properties in the solution and single crystal state

**Fig. 2:**
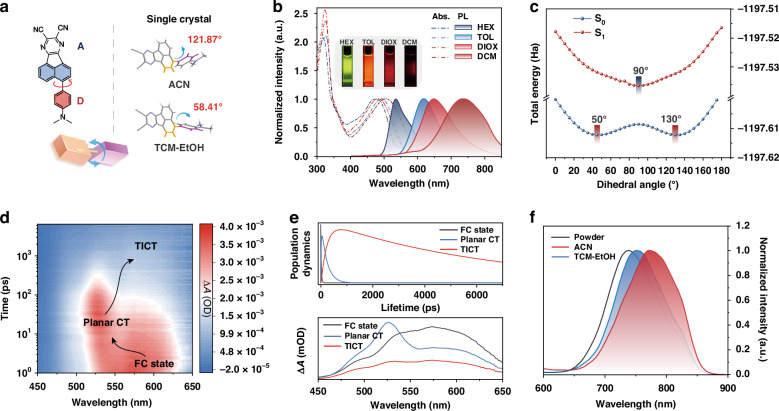
The characterization of solution-state and single-crystal state properties of CN. **a** Molecule structure of **CN** (left), single crystal structures (right) of **CN** obtained from ACN and TCM-EtOH; **b** Absorption (dotted line) and PL (solid line) spectra of **CN** in different solvents (λ_ex_ = 360 nm, C = 1×10^-4 ^mol/L, Inset: photograph of **CN** in different solvents under UV light); **c** Potential energy surface scanning of **CN** with different dihedral angles; **d** Femtosecond TA spectra of **CN** in DCM under 360 nm excitation; **e** Top and bottom panels indicate population dynamics and EADS obtained through global analysis; **f** Fluorescence spectra of **CN** powder and single crystals (λ_ex_ = 360 nm)

As shown in Fig. S1, a decent emission centered at 575 nm with yellow afterglow was found after doping compound **CK** into **BPO** matrix. The afterglow lifetime was measured to be 87.25 ms. After decorating it with *N, N*-dimethylaniline, the compound was switched from an ICT-type molecule to a TICT-type molecule (**CN**, Fig. 2a). As shown in Fig. 2b, **CN** showed a strong peak around 320 nm (assigned to π → π* transition) and relatively weak peak in the range of 400-600 nm (assigned to charge transfer transition) in the absorption spectra after being dissolved in different solvent. Similar to previous reported TICT compound, the CT absorption band exhibited distinct shift (Δλ ≈ 30 nm) with the change of solvent polarity (detailed information in Table S2). And a much more obvious shift in fluorescence spectra was observed (Δλ ≈ 200 nm) with the polarity change (n-Hexane (HEX), toluene (TOL), dioxane (DIOX), dichloromethane (DCM)). Specifically, compound **CN** exhibited a near-infrared emission centered at 734 nm in DCM as the solvent. Additionally, a weaker emission peak at 584 nm was observed in DCM as the solvent. This dual emission behavior is also a characteristic feature of TICT molecules, with the short-wavelength emission typically attributable to the planar CT state and the long-wavelength emission to the TICT state. Potential energy surface scanning result strongly supported the TICT nature of **CN**. As shown in Fig. 2c, the dihedral angle between the naphthalene segment (Acceptor) and *N, N*-dimethylaniline segment (Donor) in the optimized structure of **CN** tended to 50° or 130°. While the lowest singlet state (S_1_) tended to take a more twisty conformation with a near 90° dihedral angle between the donor and acceptor. To further investigate the TICT nature of **CN**, femtosecond transient absorption (TA) measurement was performed under 360 nm excitation in DCM (Fig. 2d). Through global fitting of the TA data, three luminescent species were identified with lifetimes of 20 ps, 216 ps, and 6.36 ns respectively (Fig. 2e). The second and third components (216 ps/6.36 ns) were found to be nearly identical to the fluorescence lifetime at 734 nm measured by time-correlated single-photon counting (TCSPC), and were thus identified as the planar CT and TICT state (Fig. S2). Through TA spectrum, a short-lived excited-state absorption (ESA) band (550–650 nm) rapidly forms under 360 nm excitation, which can be attributed to the Franck-Condon (FC) state. This evolves into a planar CT state ESA band after 20 ps, then the planar CT state transforms into a long-lived TICT state after 216 ps. Evolution-associated difference spectra (EADS) extracted from the global analysis of femtosecond TA data also exhibited this process (Fig. 2e). These findings collectively validate the TICT nature of **CN**. Moreover, single-crystal data could also confirm the twisted molecule structure. As shown in Fig. 2a, the dihedral angles between the acceptor and donor in **CN** single crystals grown from acetonitrile (ACN) and trichloromethane-ethanol (TCM-EtOH) were 121.87° and 58.41°, respectively. It can be observed that these two single crystals exhibit a mirror-image relationship, with the difference in dihedral angles primarily arising from molecular asymmetry and consistent with theoretical calculations. The fluorescence emission of the crystals has a significant redshift compared to the solution state, which may be due to generation of aggregates (Fig. 2f).

### The photophysical properties of host–guest doping system

**Fig. 3:**
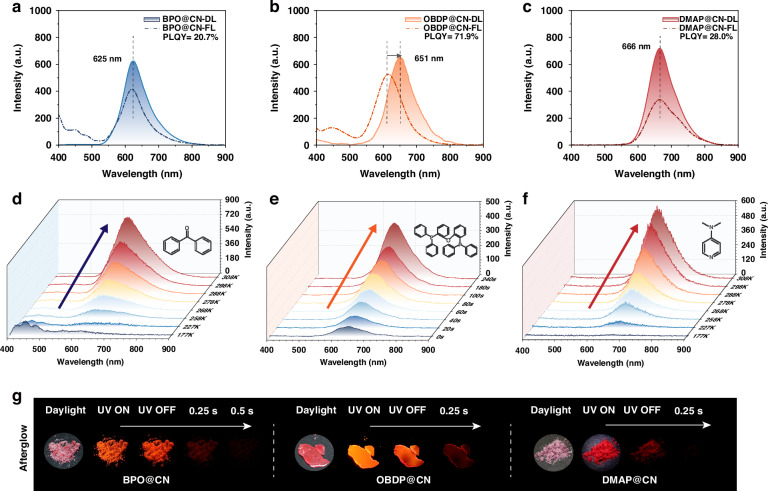
The photophysical properties of these doping materials. **a**–**c** Fluorescence (FL) and delayed (DL) spectra of **BPO**@**CN,** **OBDP**@**CN** and **DMAP**@**CN** (doping ratio 1000:1, λ_ex_ = 360 nm, delayed time = 0.5 ms); **d,** **f** Temperature-dependent delayed spectra of **BPO**@**CN** and **DMAP**@**CN**; **e** Delayed spectra of **OBDP**@**CN** at different photoactivation durations; **g** Afterglow pictures of **BPO**@**CN,** **OBDP**@**CN** and **DMAP**@**CN** (**DMAP**@**CN** excited by 310 nm and others by 365 nm)

To verify our design strategy of dopant molecules, **CN** was doped into **BPO** matrix (named as **BPO**@**CN**) with a 1000:1 doping mass ratio. As we expected, **BPO**@**CN** exhibited a distinct delayed emission around 625 nm with a lifetime of 100.32 ms and a photoluminescence quantum yield (PLQY) of 20.7% (Fig. 3a and Fig. S3). A clear orange-red afterglow lasting for more than 1.0 s could be observed by naked eyes after tuning off the 365 nm light (Fig. 3g), and this afterglow remained observable even in bright surroundings (Fig. S4). Compared with parent compound CK, the wavelength of delayed emission red-shifted about 50 nm. Furthermore, benefiting from the flexible molecule structure, the delayed emission wavelength exhibited a significant red-shift with the change of matrix. As shown in Fig. 3b, c, the delayed emission red-shifted to 651 nm, and to 666 nm with red afterglow when the matrix changed to bis(2-diphenylphosphino-phenyl) ether (**OBDP**) and 4-dimethylaminopyridine (**DMAP**) (the obtained materials named as **OBDP**@**CN** and **DMAP**@**CN**, respectively). This distinct shift (Δλ ≈ 41 nm) should be attributed to the flexible molecule structure of compound **CN**, as evidenced by the absorption spectra. As shown in Fig. S5, **BPO**@**CN** exhibited the maximum absorption wavelength at 517 nm and extended to 650 nm. The absorption spectra exhibited a distinct shift with the matrix changed to **OBDP** and **DMAP**. Specially, the maximum absorption wavelength was extended to 560 nm and tailed to 750 nm when **CN** was doped into **DMAP** matrix (Fig. S5), which implied a red-light excitability. This longer CT absorption wavelength than the solution state indicated a more twisted molecular configuration, which may result from the strong polarity and rigid micro-environment. Moreover, the fluorescence emission also exhibited a similar red-shift tendency. Among them, **DMAP**@**CN** showed the reddest fluorescence emission centered at 666 nm (Table 1). Such a redshift in absorption and emission spectra indicated that compound **CN** may take different molecule conformations in different matrices. As shown in Fig. 3b, when in **OBDP** matrix, the maximum delayed emission wavelength exhibited distinct redshift compared with the fluorescence emission. The energy difference between the delayed and fluorescence emission of **OBDP**@**CN** was calculated to be 0.08 eV, according to the emission spectra. More importantly, the lifetime of the delayed emission showed a power law decay pattern (Fig. S3b), which is the most typical character of long persistent luminescence (LPL)^42,44–47^. Furthermore, long-lived radical signals were detected in electron paramagnetic resonance (EPR) experiment (Fig. S6). Therefore, the delayed emission of **OBDP**@**CN** could be assigned to the long-lived emission from exciplex. Both temperature-dependent spectra and exciton accumulation phenomena can well support this viewpoint (Figs. S7 and 3e). Similarly, power law decay was found by doping CK into **OBDP** (Fig. S8). When the matrix was changed to **BPO** or **DMAP**, the delay emission, with a lifetime of 100.32 ms and 47.82 ms, respectively (Fig. S3), showed a same spectrum with the fluorescence emission, indicating the delayed emission was likely to be from the singlet excited state. More importantly, as the temperature decreasing, the delayed emission intensity kept decreasing, and the temperature-dependent lifetime decay curve exhibited a non-monotonic trend. As the temperature rising, the lifetime first increased and then decreased (Figs. 3d, f and S9). These phenomena indicated that the afterglow of **BPO**@**CN** and **DMAP**@**CN** was likely be thermally activated delayed fluorescence (TADF) type afterglow^48–51^.

**Table 1:** The key photophysical parameters of **BPO**@**CN,** **DMAP**@**CN,** **OBDP**@**CN** (doping ratio = 1000:1)

	λ_Abs_ (nm)	λ_Ex_ (nm)	λ_F_ (nm)	λ_DF_ (nm)	τ (ms)	PLQY
**BPO**@**CN**	527	560	625	625	100.32	20.7%
**DMAP**@**CN**	517	631	666	666	47.82	28.0%
**OBDP**@**CN**	560	544	623	651	/	71.9%

“/” indicates that the lifetime of the LPL is not calculated

### The visible light excitation capability of these doping materials

**Fig. 4:**
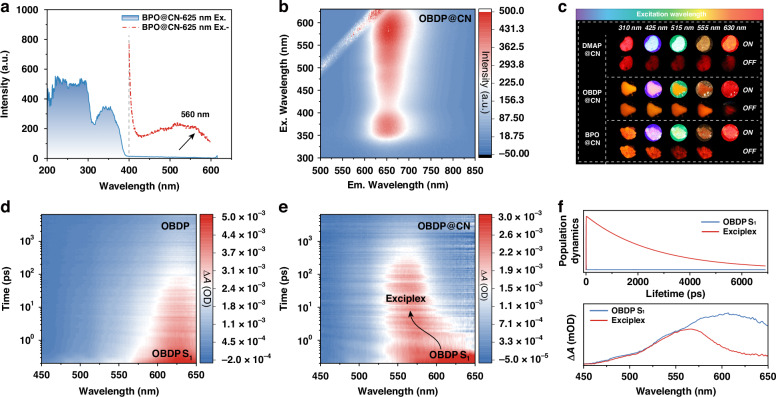
The visible light excitation capability and intrinsic mechanism of dynamic afterglow. **a** Excitation spectra of **BPO**@**CN**; **b** Emission-Excitation dependent spectra of **OBDP**@**CN**; **c** Afterglow photographs of **BPO**@**CN,** **OBDP**@**CN** and **DMAP**@**CN** under different excitation wavelength; **d**, **e** Femtosecond TA spectra of **OBDP** and **OBDP**@**CN** under 360 nm excitation; **f** Top and bottom spectra indicate population dynamics and EADS of **OBDP**@**CN** obtained through global analysis

More importantly, the excitation spectra of all materials extended to visible light region. As shown in Fig. 4a, **BPO**@**CN** showed a strong excitation peak in the range of 200–400 nm, which is assigned to the excitation band of **BPO**. While a relatively weak excitation band was also recorded in the range of 400–600 nm, which is assigned to the excitation of **CN**. Furthermore, excitation-emission dependent spectra further demonstrated the full-spectrum excitation capability of these materials (Fig. S10). However, under long-wavelength excitation, the delayed emission peak exhibited a redshift compared to that under short-wavelength excitation, which may be attributed to the conformational diversity of the **CN** molecules. Similar phenomena were also observed for **OBDP**@**CN** and **DMAP**@**CN**, except that **OBDP**@**CN** exhibited relatively strong excitation efficiency in the visible light region (Figs. 4b, S11 and S12). Such long excitation wavelength should originate from the decreased energy gap between the HOMO and LUMO of **CN** by CT effect. Figure 4c showed the afterglow pictures of **BPO**@**CN,** **DMAP**@**CN** under different excitation wavelengths. It’s worth noting that, **DMAP**@**CN** showed a maximum peak around 631 nm in the excitation spectra and tailed to 650 nm, which is the longest excitation wavelength for afterglow materials (Fig. 1c). The clear red afterglow could even be observed under 630 nm flashlight excitation (Fig. 4c).

### The intrinsic mechanism of dynamic afterglow

Exciplex is the most convincible mechanism for such bi-component afterglow materials. According to this mechanism, it is considered that an exciplex will form after excitation^52–54^. Then, a long-lived emission is generated either from the exciplex or from the triplet excited state of the dopant. The prerequisite for the generation of exciplex is that the energy levels of the HOMO and LUMO of host and guest should match a type II alignment. However, **BPO** does not meet this prerequisite (Fig. S13). **BPO** is a famous phosphor with efficient ISC yield. Our previous work indicated that the interaction between **BPO** and the dopant occurs at the triplet state^55^. Therefore, the singly occupied molecular orbitals (SOMOs) of the host material **BPO** are introduced into the frontier orbital energy diagrams of **CN** to gain further insight. Based on the T_1_ energy, the SOMO energy level of **BPO** can be estimated to be about -3.87 eV. The energy levels of HOMO and SOMO of **BPO** and HOMO and LUMO of **CN** match a type-II arrangement, which allows the formation of exciplex^56^. To further elucidate the mechanism of exciplex formation, femtosecond TA spectra were acquired for both host-guest doped materials (**BPO**@**CN,** **OBDP**@**CN,** **DMAP**@**CN** and **PMMA@CN**) and pure hosts (**BPO,** **OBDP** and **DMAP**) (Figs. 4d–f and S14–S19). Taking the **OBDP**@**CN** doping system as an example, as shown in Fig. 4d, e, neither ground-state bleaching (GSB) signals nor stimulated emission (SE) signals were detected in the TA spectra of either **OBDP**@**CN** or **OBDP**. Through global fitting of TA data, a new luminescent species was observed in **OBDP**@**CN**. (Fig. 4f). Both **OBDP**@**CN** and **OBDP** exhibited similar ESA signals in the 600-650 nm range immediately after excitation, attributable to the absorption of the **OBDP** singlet excited state. In **OBDP**, the signal at 625 nm decayed over 7 ns. However, in **OBDP**@**CN**, the 625 nm signal decayed to zero within 10 ps, followed by the emergence of a new long-lived ESA signal at 562 nm. This can be attributed to the signal of the exciplex. This peak is entirely distinct from the signal peak (542 nm) observed in **PMMA@CN** (Fig. S15). The population dynamics provided a more intuitive representation of the evolutionary process of the luminescent species (Fig. 4f). These findings indicate complex interactions between **OBDP** and **CN**, leading to the generation and evolution of new species which exhibit distinct photophysical properties. Owing to the difficulty in producing a sufficiently uniform and transparent film of **DMAP**@**CN**, its TA spectral quality was suboptimal. Nevertheless, we can still observe an emission process analogous to that of **OBDP**@**CN** (Fig. S16). Slightly different, **BPO** and **BPO**@**CN** appeared to possess similar TA spectra. However, global fitting analysis revealed a new species generated from the **BPO** triplet state within the **BPO**@**CN**, which can be classified as the exciplex (Figs. S17–S19)^55^. Fig. S20 showed a simplified diagram of the mechanistic process. It could be inferred from the excitation spectra that the excitation of either host or guest could produce exciplex. For **BPO**@**CN** and **DMAP**@**CN**, the exciton went from the excited state of the exciplex returned to the singlet or triplet of **CN**, and was finally emitted as TADF. Whereas in the **OBDP**@**CN** system, LPL arose from the charge separation and recombination of exciplex. PLQY and lifetime under different excitation wavelengths further substantiated this conjecture, with PLQY and lifetime remained virtually identical regardless of whether long-wavelength or short-wavelength excitation was employed (Figs. S21, S22 and Table S3). **CN** exhibited different delayed emissions in various matrices, likely due to its flexible molecular skeleton. This flexibility allows **CN** to adopt twisted but distinct molecular configurations in different environments, as supported by the absorption spectra. Consequently, the delayed emission wavelength of **CN** varied across matrices.

To further validate the mechanism of dynamic afterglow, two methyl groups were introduced to the compound **CN** to restrain the rotation of *N, N*-dimethylaniline group (**EN**). As shown in Fig. S23, *N, N*-dimethylaniline group is almost orthogonal to the naphthalene segment in the optimum structure. The dihedral angle between them in the ground state was calculated to be about 89.85°. PES scan result showed compound **EN** tend to take an orthogonal molecular configuration both in S_0_ and S_1_ state (Figs. S24 and S25). *N, N*-dimethylaniline group was almost unable to twist because of the steric hindrance. Absorption spectra of **EN** in different solvents could also confirmed this point. Similar to **CN,** **EN** showed a strong absorption peak around 320 nm (assigned to π → π* transition) in different solvents (Fig. S26). And the absorbance of the CT absorption band (400–600 nm) significantly decreased compared with compound **CN**, which may indicate the charge transfer between donor and acceptor was blocked. Compound **EN** showed a very similar delayed emission around 605 nm when doped into **BPO,** **OBDP** or **DMAP** matrix (Fig. S27 and S28). These very similar delayed emission wavelengths testified the variable delay emission of **CN** resulted from the molecular structural flexibility. As we mentioned above, TADF is the basis for such rich photophysical properties of **CN**. To further testify it, compounds **CN** was doped into polymethyl methacrylate (PMMA) matrix. As shown in Fig. S29, the obtained film (**PMMA@CN**) showed similar delayed emission spectrum (τ = 0.67 ms) with the fluorescence, and both its luminescence intensity and lifetime exhibited a trend of first increasing and then decreasing with rising temperature (Fig. S30), proving the TADF property of **CN**^57–59^. The slight redshift of the delayed emission may result from the restraint of the rotation in the rigid micro-environment. These phenomena jointly prove our hypothesis.

### Photophysical properties of near-infrared afterglow

**Fig. 5:**
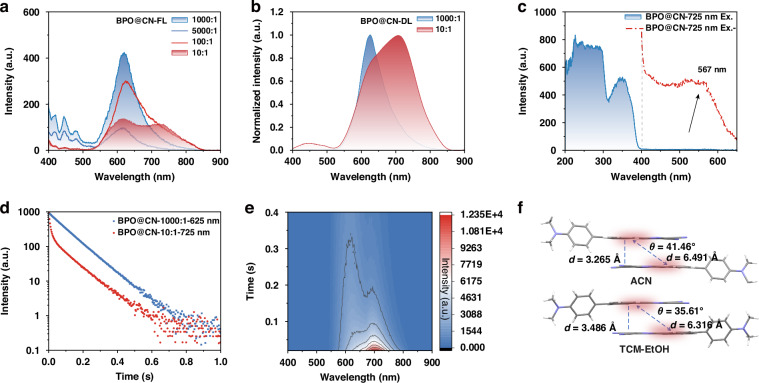
Photophysical properties of near-infrared afterglow. **a** FL and **b** DL spectra of **BPO**@**CN** under different doping ratios; **c** Excitation spectra of **BPO**@**CN** under doping ratio 10:1; **d** Lifetime decay curves and **e** Time-resolved spectra of **BPO**@**CN** (doping ratio 10:1); **f** Single crystal stacking diagram of ACN and TCM-EtOH

During our research, we found **BPO**@**CN** showed an interesting concentration dependence. As shown in Fig. 5a, **BPO**@**CN** showed a maximum emission at 625 nm in the fluorescence spectrum when the doping ratio was set as 5000:1. The emission showed obvious increase and maintained in similar shape with the doping ratio raised to 1000:1. A shoulder peak in the range of 700–800 nm was generated when the ratio was further raised to 100:1. And the emission intensity around 625 nm showed slight decrease compared with 1000:1. The shoulder peak evolved to a distinct emission peak at 725 nm with the ratio increased to 10:1. Similar variation, which further confirmed the TADF nature of the delay emission, could also be observed in the delayed emission spectra (Fig. 5b). Particularly, the maximum excitation wavelength extended to 567 nm and tailed to 700 nm (Fig. 5c). The lifetime decay curve reveals that the lifetime at 725 nm (τ = 67.82 ms, Fig. 5d) exhibited two distinct phases: a rapid decay prior to 0.1 s, followed by a slow decay thereafter. Time-resolved spectra further corroborated this lifetime decay behavior at 725 nm (Fig. 5e). It was crucial to emphasize that the long lifetime observed at 725 nm is genuinely present and does not originate from the 625 nm component. The newly emerged emission peak at 725 nm was speculated to be the emission from J-aggregation, which can be evidenced by single crystal data^60^. Single-crystal stacking structure of **CN** indicated the formation of J-aggregates (Fig. 5f), which supports our speculation. Similar delayed emission from J-aggregation could also be observed in the **DMAP** and **OBDP** matrices (Fig. S31).

### In vivo afterglow bioimaging application

**Fig. 6:**
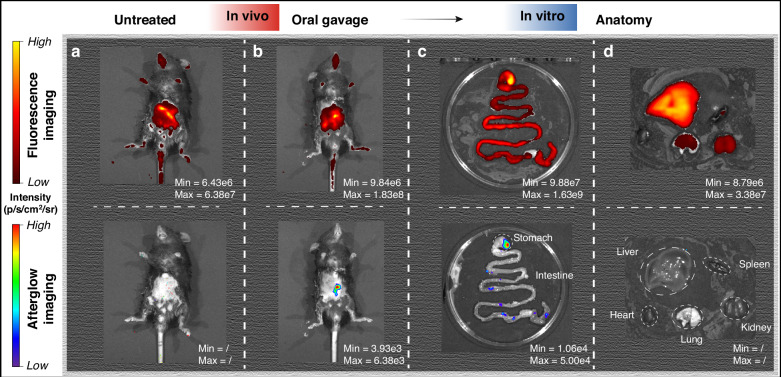
In vivo afterglow bioimaging application. In vivo fluorescence and afterglow bioimaging of the **a** untreated and **b** oral gavage in mice; In vitro (**c**) and (**d**) fluorescence and afterglow bioimaging of mice after anatomy (fluorescence imaging signals displayed in ‘YellowHot’ color table, afterglow imaging signals displayed in “Rainbow” color table)

Afterglow imaging holds potential for eliminating interference from tissue autofluorescence signals in imaging applications due to its long-lived luminescence properties. Consequently, we formulated the near-infrared afterglow material developed in this study into a contrast agent to validate its afterglow imaging capabilities. The contrast agent was administered to mice via oral gavage. This contrast agent comprised **BPO**@**CN**, DSPE-mPEG (molecular weight 2000), gum Arabic, and phosphate-buffered saline. DSPE-mPEG serves as a surfactant, whilst gum Arabic is a plant-derived thickening polysaccharide. Using the bioluminescence mode of an in vivo imaging system (IVIS), we imaged the mouse stomach using the afterglow following red light irradiation. As shown in Fig. S34, the contrast agent sustained detectable afterglow signals for over 15 min after light irradiation cessation. Following administration of the contrast agent, the stomach signals in mice were difficult to visualize effectively in fluorescence mode due to interference from the liver, intestine and tissue autofluorescence. However, in bioluminescence mode, obvious signals originating from the stomach were observed using afterglow imaging, and no effective signal was observed for untreated mice (Fig. 6a, b). To further confirm whether the afterglow signals originated from the stomach, we performed dissections on the mice. As shown in the Fig. 6c, d, fluorescence mode revealed distinct signals in the mouse gastrointestinal tract and liver regions, distinguishing these signals from the contrast agent versus tissue autofluorescence was proved challenging, hindering precise pathological information acquisition. In contrast, afterglow imaging generated distinct signals in the stomach under bioluminescence mode, eliminating fluorescence interference and facilitating enhanced pathological assessment within living organisms. This study demonstrates the potential of visible light-excited near-infrared afterglow contrast agents as highly effective in vivo biosensing tools. They significantly optimize response performance while effectively suppressing autofluorescence interference.

## Discussion

In conclusion, we proposed a molecular design strategy toward visible-light-excited organic afterglow materials by modifying an electron-deficient dopant with electron-donating groups. By doping the obtained TICT-type dopant (**CN**) into different matrices, a series of red-light-excited NIR afterglow materials was obtained, with various performance (decay path, lifetime emission, emission wavelength). Such rich photophysical property variations were mainly attributed to the twisted molecular structure of **CN**. It was worth mentioning that a NIR afterglow (τ = 67.82 ms) centered at 725 nm was obtained. Moreover, the excitation wavelength of the afterglow was extended to 650 nm. By the afterglow imaging technology of utilizing these long-wavelength-excitable near-infrared afterglow materials, the critical challenge of tissue autofluorescence interference in conventional fluorescence imaging was successfully resolved.

## Materials and methods

### Synthesis

The synthesis route to the target compounds and the experimental details on the synthesis are provided in the Supplementary Information.

### Characterization

^1^H NMR and ^13^C NMR spectra were measured on a Bruker AV-600 spectrometer. The electronic spray ionization (ESI) high-resolution mass spectra were tested on a Waters LCT Premier XE spectrometer. The UV–Vis absorption spectra and PL spectra were performed on a Varian Cray 500 spectrophotometer. Fluorescence, phosphorescence, and lifetime of delayed emission spectra were recorded on an Agilent Cary Eclipse spectrophotometer. Fluorescence lifetimes were measured using the TCSPC system of the FLS1000 fluorescence spectrometer from Edinburgh Instruments, in conjunction with the EPL-375 tunable laser. Absolute PL quantum yields were determined with a spectrometer C11347-11 (Hamamatsu, Japan). Femtosecond transient absorption spectra were acquired by using the Time-Tech Spectra TA100 instrument. Afterglow bioimaging was conducted by using the PerkinElmer IVIS Spectrum CT imaging system. The animal procedures were conducted in strict accordance with the Guidelines for Care and Use of Laboratory Animals of East China University of Science and Technology. The compounds and solvents used in the synthesis were purchased from Adamas-beta.

### Theoretical methods

Density functional theory (DFT) and time-dependent (TD-DFT) calculations were performed with the Gaussian 16 (Revision E.01) software package. The ground-state (S_0_) geometries were optimized with the B3LYP and 6-311G* basis. The excitation energies in the singlet and triplet states were obtained using TD-DFT method based on the optimized S_0_ molecule structure.

## Supplementary information

Supplementary information.docx

## Data Availability

The authors declare that the data supporting the findings of this study are available within the article and its Supplementary Information file. All data are available from the authors upon reasonable request.
