# Supplementary material for: Red-light-excited dynamic near-infrared organic afterglow materials for in vivo bioimaging
Source: Light Sci Appl. 2026 Jun 10;15:271. doi: 10.1038/s41377-026-02340-3 (PMC13254300; doi:10.1038/s41377-026-02340-3)
Supplement: Supplementary file 1 — Supplementary information.docx [file 41377_2026_2340_MOESM1_ESM.docx]

Supplementary Information for

**Red-light-excited dynamic near-infrared organic afterglow materials for *in vivo* bioimaging**

**Lei Zhou, Jiacheng Yang, Zhenyi He, Zhiqin Wu, Ping Jiang, Jinming Song, Liangwei Ma*, He Tian, and Xiang Ma***

Key Laboratory for Advanced Materials and Feringa Nobel Prize Scientist Joint Research Center, Frontiers Science Center for Materiobiology and Dynamic Chemistry, Institute of Fine Chemicals, School of Chemistry and Molecular Engineering, East China University of Science and Technology, Meilong Road 130, Shanghai 200237, P. R. China. Email: [maxiang@ecust.edu.cn](mailto:maxiang@ecust.edu.cn), [liangweima@ecust.edu.cn](mailto:liangweima@ecust.edu.cn)

Synthesis route of **CK**：


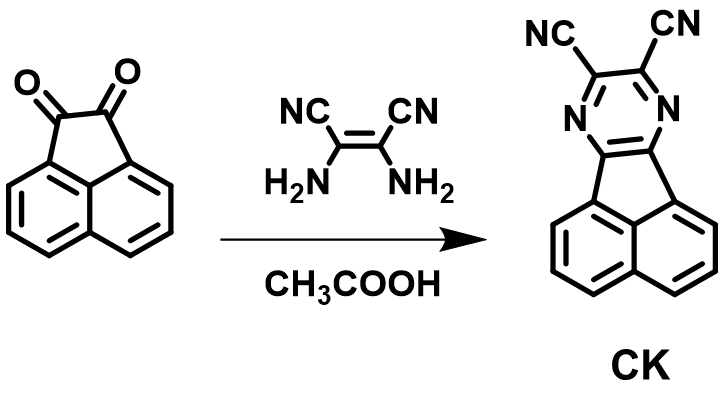


Compound CK was synthesized with reference to previous literature.^[1]^

1.00 g (5.49 mmol, 1 eq) of intermediate product and 0.59 g (5.49 mmol, 1 eq) of diaminomaleonitrile anhydride were dissolved in acetic acid (80 mL). The reaction mixture was stirred overnight at 115℃ under nitrogen atmosphere. The reaction was quenched with water, filtered to get the product and purified by column chromatography on silica (DCM) to give CK (yellow solid) 1.23 g. Yield: 88.16%.

^1^H NMR (600 MHz, CDCl_3_, *δ*) 8.55 (d, *J* = 6.0 Hz, 2H), 8.34 (d, *J* = 6.0 Hz, 2H), 7.99 - 7.96 (dd, *J* = 6.0 Hz, 2H). ^13^C NMR (151 MHz, CDCl_3_) *δ* 154.73, 132.83, 129.94, 129.78, 129.43, 128.63, 126.28, 122.13, 114.19. HRMS (DART) (m/z): [M+H]^+^ cacl. for [C_16_H_7_N_4_]^+^, 255.0665; found, 255.0650.

Synthesis route of **CN**：


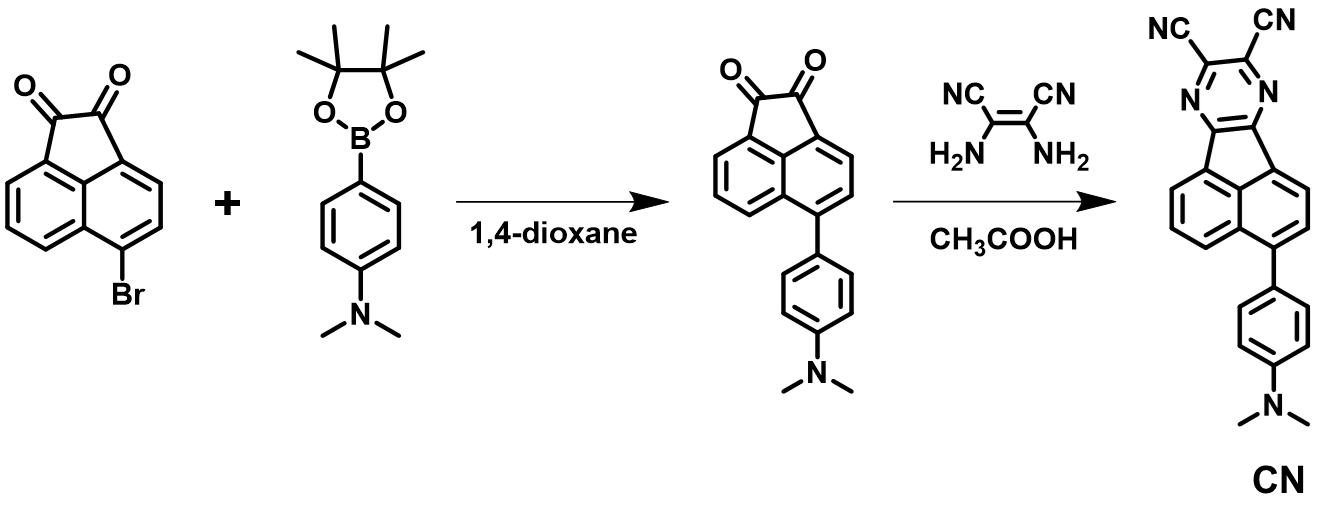


5-Bromoacenaphthenequinone (2.00 g, 7.66 mmol, 1 eq), *N, N*-Dimethyl-4-(4,4,5,5-tetramethyl-1,3,2-dioxaborolan-2-yl)aniline (2.27 g, 9.20 mmol, 1.2 eq), Potassium carbonate (3.17 g, 23 mmol, 3 eq) and Tetrakis(triphenylphosphine)palladium (0.38 g, 0.38 mmol, 0.05 eq) were added to 1,4-Dioxane (100 mL) solvent and deionized water (25 mL). The reaction mixture was stirred overnight at 120℃ under nitrogen atmosphere. The reaction was quenched with water, extracted with DCM, dried over MgSO_4_, filtered, concentrated, and purified by column chromatography on silica (DCM: PE = 3: 1) to give intermediate product (dark red solid) 1.56 g. Yield: 67.7%. 1.00 g (3.32 mmol, 1 eq) of intermediate product and 0.36 g (3.32 mmol, 1 eq) of diaminomaleonitrile anhydride were dissolved in acetic acid (80 ml). The reaction mixture was stirred overnight at 115℃ under nitrogen atmosphere. The reaction was quenched with water, filtered to get the product and purified by column chromatography on silica (DCM: MeOH = 50: 1) to give CN (dark purple solid) 1.04 g. Yield: 84.02%.

^1^H NMR (600 MHz, CDCl_3_, *δ*) 8.55 - 8.54 (t, *J* = 6.0 Hz, 2H), 8.49 (d, *J* = 6.0 Hz, 1H), 7.94 (dd, *J* = 6.0 Hz, 1H), 7.90 (d, *J* = 6.0 Hz, 1H), 7.60 (d, *J* = 6.0 Hz, 2H), 7.04 (s, 2H), 3.13 (s, 6H). ^13^C NMR (151 MHz, CDCl_3_) *δ* 154.71, 145.89, 135.24, 133.46, 132.81, 132.64, 131.02, 129.91, 129.75, 129.40, 128.61, 128.59, 128.47, 126.26, 122.10, 118.90, 114.16, 29.68. HRMS (DART) (m/z): [M+H]^+^ cacl. for [C_24_H_16_N_5_]^+^, 374.1400; found, 374.1400.

Synthesis route of **EN**：


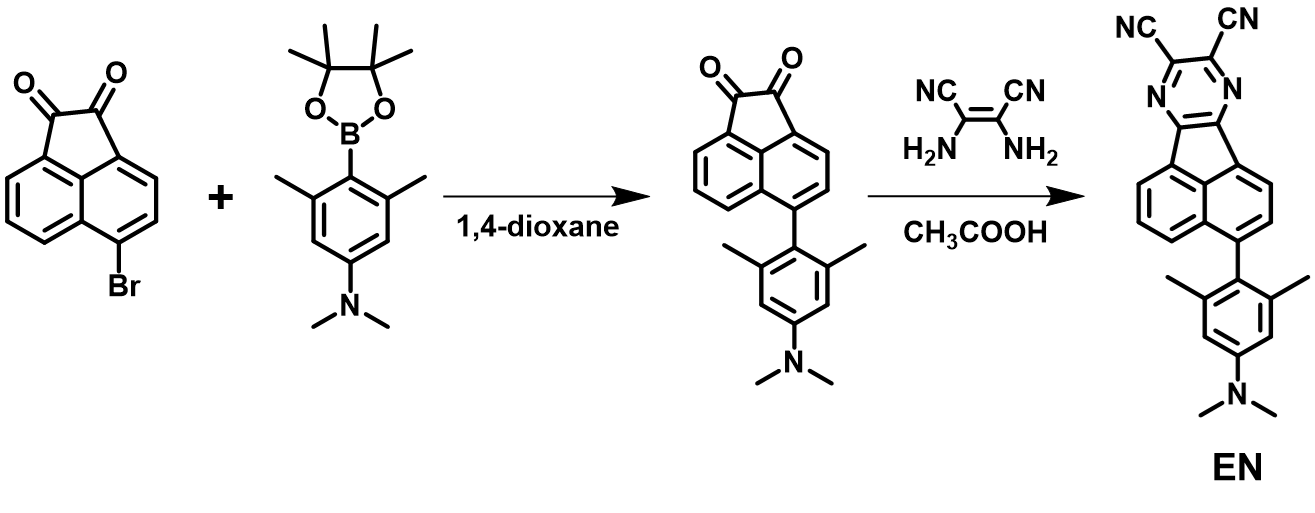


5-Bromoacenaphthenequinone (0.16 g, 0.61 mmol, 1 eq), *N, N*,3,5-tetramethyl-4-(4,4,5,5-tetramethyl-1,3,2-dioxaborolan-2-yl)aniline (0.20 g, 0.73 mmol, 1.2 eq), Potassium carbonate (0.25 g, 1.83 mmol, 3 eq) and Tetrakis(triphenylphosphine)palladium (0.03 g, 0.03 mmol, 0.05 eq) were added to 1,4-Dioxane (10 mL) solvent and deionized water (2 mL). The reaction mixture was stirred overnight at 120℃ under nitrogen atmosphere. The reaction was quenched with water, extracted with DCM, dried over MgSO_4_, filtered, concentrated, and purified by column chromatography on silica (DCM: PE = 3: 1) to give intermediate product (dark red solid) 10.27 mg. Yield: 5.12%. 0.05 g (0.15 mmol, 1 eq) of intermediate product and 16.20 mg (0.15 mmol, 1 eq) of diaminomaleonitrile anhydride were dissolved in acetic acid (10 mL). The reaction mixture was stirred overnight at 115 ℃ under nitrogen atmosphere. The reaction was quenched with water, filtered to get the product and purified by column chromatography on silica (DCM: MeOH = 50: 1) to give EN (dark purple solid) 27.12 mg. Yield: 45.08%.

^1^H NMR (600 MHz, CDCl_3_, *δ*) 8.56 (d, *J* = 6.0 Hz, 1H), 8.52 (d, *J* = 6.0 Hz, 1H), 7.90 (d, *J* = 6.0 Hz, 1H), 7.84 (d, *J* = 6.0 Hz, 1H), 7.75 (d, *J* = 6.0 Hz, 1H), 6.62 (s, 2H), 3 3.05 (s, 6H), 1.93 (s, 6H). ^13^C NMR (151 MHz, CDCl_3_) *δ* 153.68, 149.39, 146.56, 136.14, 134.70, 130.92, 130.06, 128.78, 128.39, 128.16, 127.69, 126.07, 125.33, 125.06, 124.13, 113.30, 110.48, 39.46, 20.28. HRMS (DART) (m/z): [M+H]^+^ cacl. for [C_26_H_20_N_5_]^+^, 402.1713; found, 402.1689.

**Preparation of the doped materials:** Guest and Host were mixed homogeneously by sonication and then the mixture was heated to a melting point to form a molten mixture. The molten mixture was cooled at room temperature to obtain the dopant material. For example, BPO@CN (doping ratio 1000:1): 1 mg of CN and 1000 mg of benzophenone (BPO) were mixed homogeneously by sonication and then the mixture was heated to 50°C to form a molten mixture. The molten mixture was cooled at room temperature to obtain a solidified fused sample, named as BPO@CN. The processing temperature for OBDP@CN and DAMP@CN is 185°C and 120°C.

**Preparation of the single crystals:** The single crystal of ACN was obtained by dissolving CN in acetonitrile solvent, and slowly evaporating the solvent. The single crystal of TCM-EtOH was obtained by dissolving CN in deuterated chloroform and ethanol (1: 1) solvent, and slowly evaporating the solvent. CCDC numbers: 2539391 (ACN) and 2539395 (TCM-EtOH)

**Preparation of the contrast agent:** The contrast agent is a colloidal suspension composed of BPO@CN, DSPE-mPEG (molecular weight 2000), gum Arabic, and phosphate-buffered saline. To 2 mL of the phosphate-buffered saline solution of DSPE-mPEG (molecular weight 2000, 20 mg) and gum Arabic (8 mg), the BPO@CN crystals (2 mg) were added. The mixture was then sonicated by a microtip-equipped probe solicitor (XM-1000DT) for 10 min. Allow the resulting solution to stand for half an hour, then aspirate the middle layer to obtain the colloidal suspension (DLS data in Fig. S32).

***In vivo* afterglow imaging:** The images were acquired after removal of 630 nm handheld red lamp (10 W) using IVIS system under the bioluminescence mode with open filter setting. Quantification of afterglow and fluorescence was performed by ROI analysis of the interest region using Living Imaging software version 4.4. Pentobarbital sodium (3 wt.% in saline solution) was used as the anesthetic.

**Cell culture and cytotoxicity experiments:** The HeLa cells were cultured in Gulbecco's modified Eagle medium (DMEM) supplemented with 10% fetal bovine serum (FBS) and 1% penicillin/streptomycin, at a humidified atmosphere with 5% CO_2_ at 37°C. In vitro cytotoxicity of the nanocrystals against cancer cells was assessed by 3-(4,5dimethylthiazol-2-yl)-2,5-diphenyl tetrazolium bromide (MTT) assay. HeLa cancer cells seeded in 96-well plates were exposed to each kind of nanoparticles concentrations of 12.5, 25, 50, 100, 200, 300, 400 μg·mL^−1^, respectively, at 37 °C. After 24 h incubation, the wells were washed twice with PBS, and 100 μL of freshly prepared MTT (0.5 mg·mL^−1^) solution in culture medium was added into each well. The MTT medium solution was carefully removed after 4 h incubation in the incubator. DMSO (150 μL) was then added into each well and the plate was gently shaken for 10 min at room temperature to dissolve all the precipitates formed. The absorbance of MTT at 490 nm was monitored by the microplate reader (Genios Tecan). Cell viability was expressed by the ratio of absorbance of the cells incubated with nanoparticles suspension to that of the cells incubated with culture medium only (biotoxicity assessment data in Fig. S33).

**Table S1.** Reported visible-light excited afterglow materials.

| Sample name | Excitation wavelength (nm) | Emission wavelength (nm) |
| --- | --- | --- |
| PCD@BA^[2]^ | 450 | 535 |
| Cor/HIPS^[3]^ | 500 | 567 |
| 9PBA@PVA1799^[4]^ | 450 | 520 |
| a-DThBSS-Cz^[5]^ | 457 | 577 |
| CBtCOONa^[6]^ | 400 | 572 |
| BA@Fluo^[7]^ | 460 | 483 |
| TpPBr^[8]^ | 490 | 610 |
| CzEL^[9]^ | 433 | 501 |
| CzPL^[9]^ | 417 | 553 |
| Br-NpCzBF^[10]^ | 470 | 647 |
| TRZ-4^[11]^ | 480 | 590 |
| t-DTBT^[12]^ | 480 | 660 |
| BrPmDI^[13]^ | 450 | 650 |
| m,p/CDs-ME^[14]^ | 500 | 508 |
| C4-Br^[15]^ | 450 | 607 |
| CalNa-Al_2_(SO4)_3_^[16]^ | 450 | 584 |
| CPhCz^[17]^ | 410 | 530 |
| CzBH^[18]^ | 420 | 545 |
| NDI-Br-Ph-PLA 400^[19]^ | 530 | 700 |
| CP4^[20]^ | 498 | 732 |

**Table S2**. Photophysical data of the CN in different solvents.

| Solvent | Absorption wavelength (nm) | Emission wavelength (nm) | Stokes shift (nm) |
| --- | --- | --- | --- |
| HEX | 475 | 534 | 59 |
| TOL | 484 | 615 | 131 |
| DIOX | 491 | 646 | 155 |
| DCM | 504 | 734 | 230 |


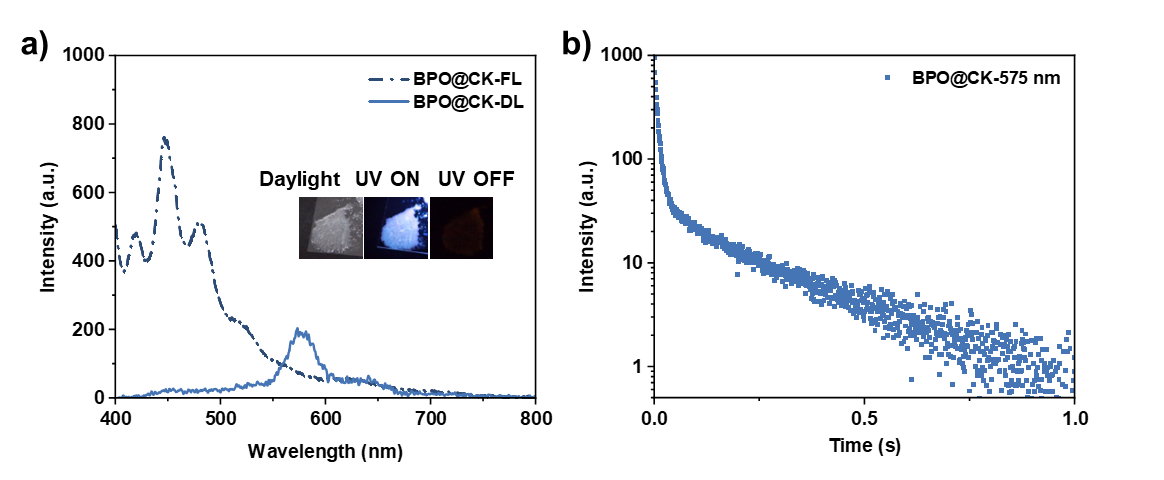


**Fig. S1.** a) The prompt, delayed spectra (illustration: afterglow pictures) and b) lifetime decay curves of BPO@CK (doping ratio 1000: 1, λ_ex_ = 360 nm, delayed time = 0.5 ms)


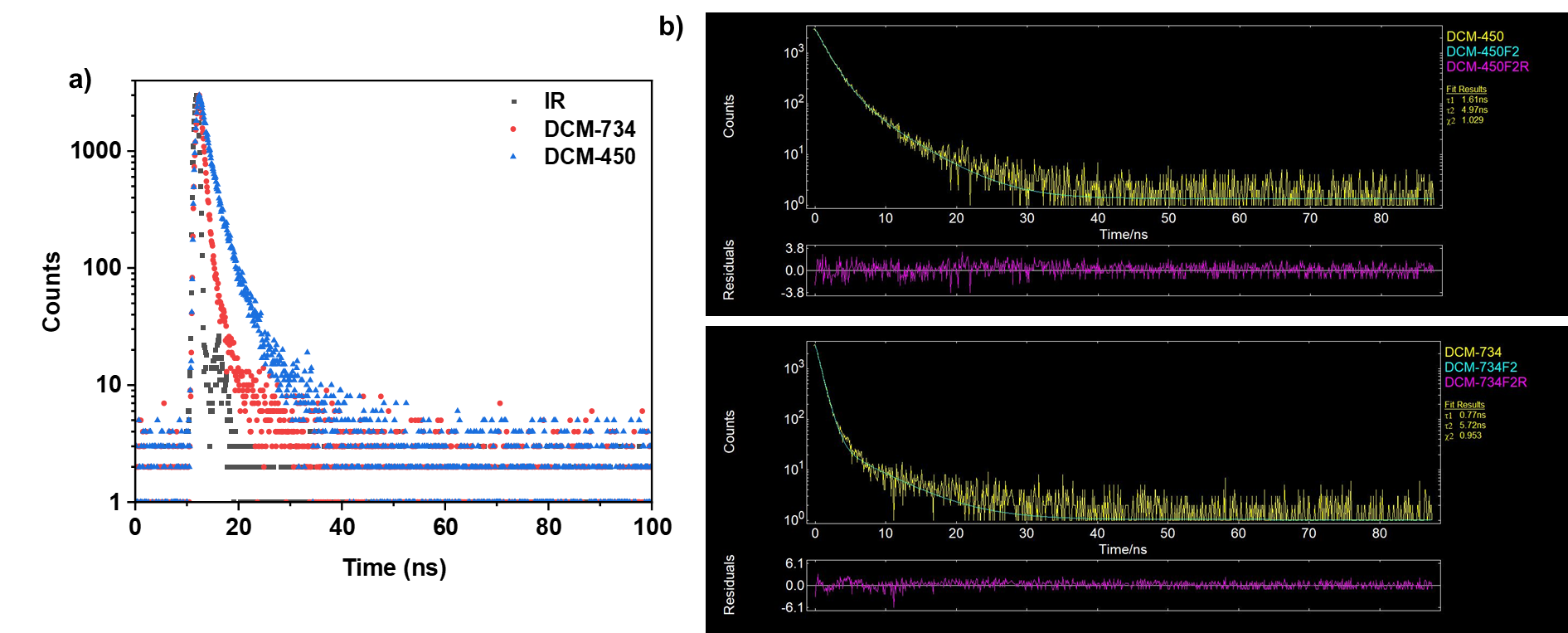


**Fig. S2.** a) and b) The FL lifetime decay curves of CN in DCM (λ_ex_ = 375 nm, C = 1×10^-4^ mol/L)


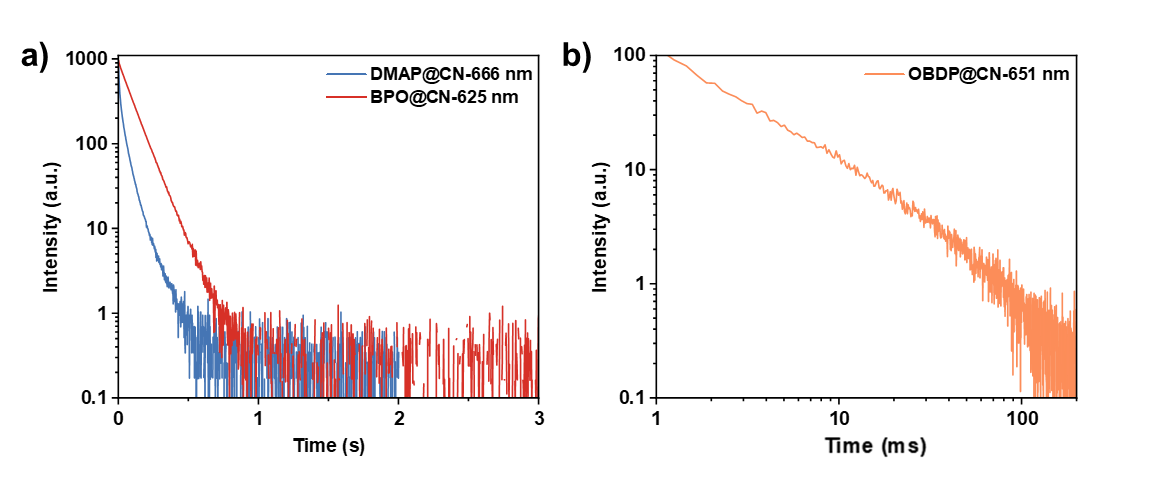


**Fig. S3.** a) and b) Lifetime decay curves of BPO@CN, OBDP@CN and DMAP@CN.


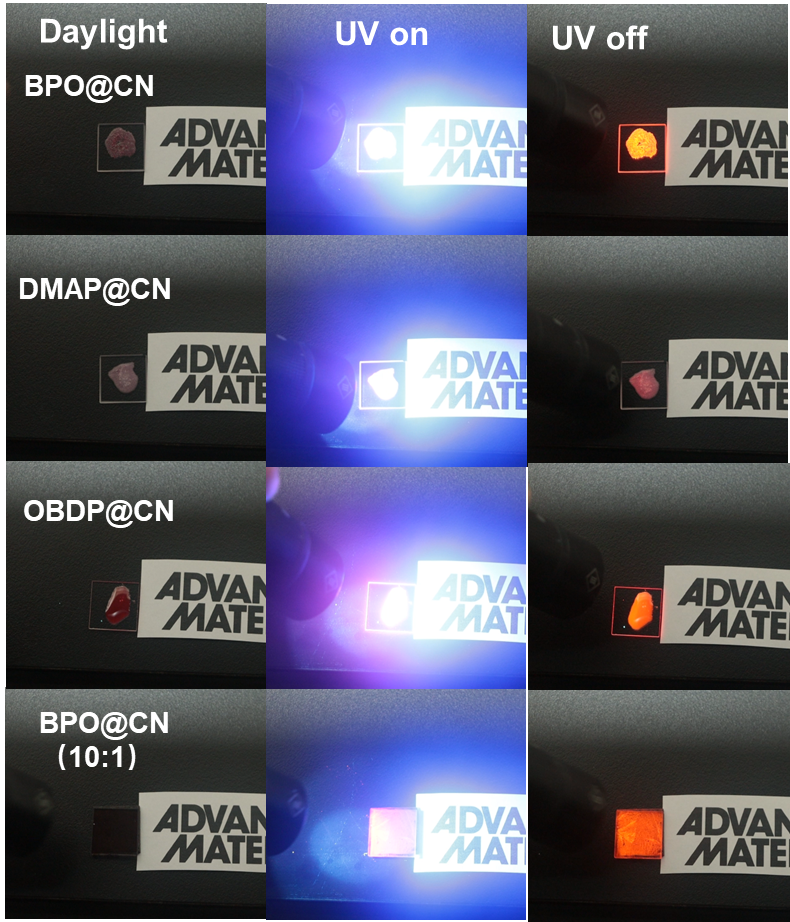


**Fig. S4.** Afterglow of BPO@CN, OBDP@CN, DMAP@CN.


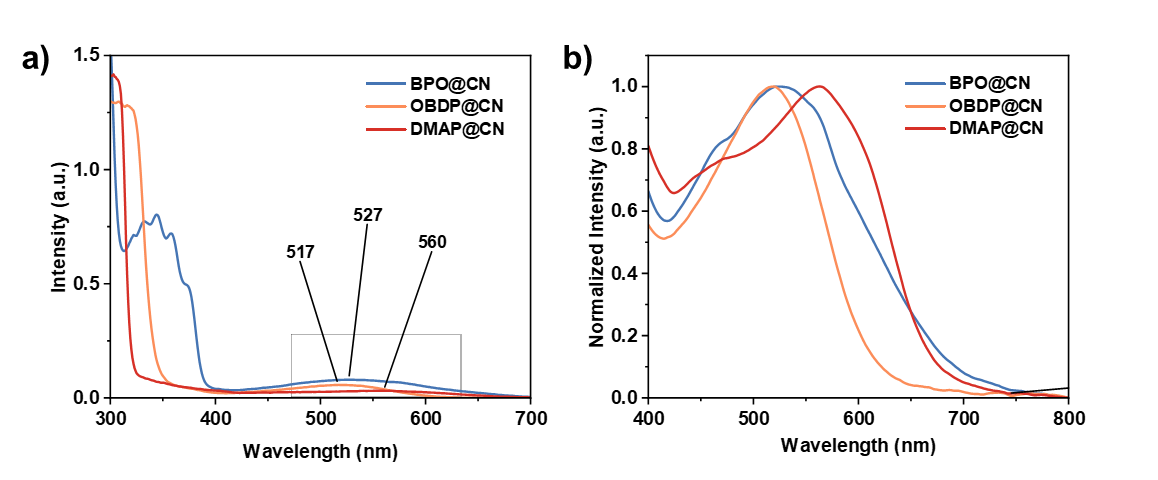


**Fig. S5.** a) Absorption spectra and b) normalized absorption spectra of BPO@CN, OBDP@CN and DMAP@CN.


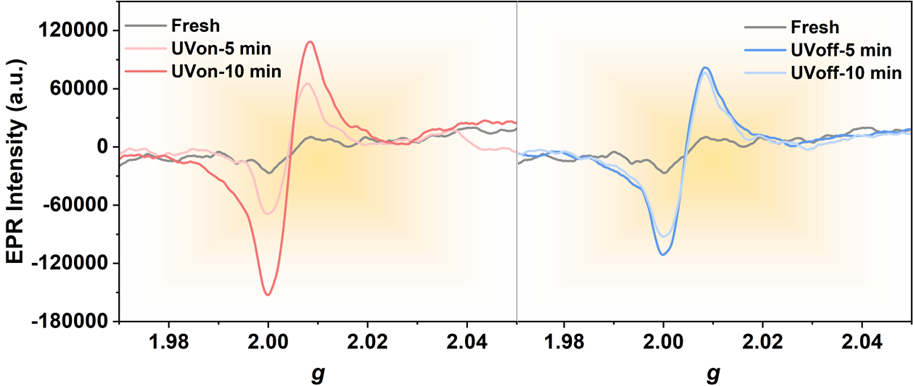


**Fig. S6.** EPR of OBDP@CN.


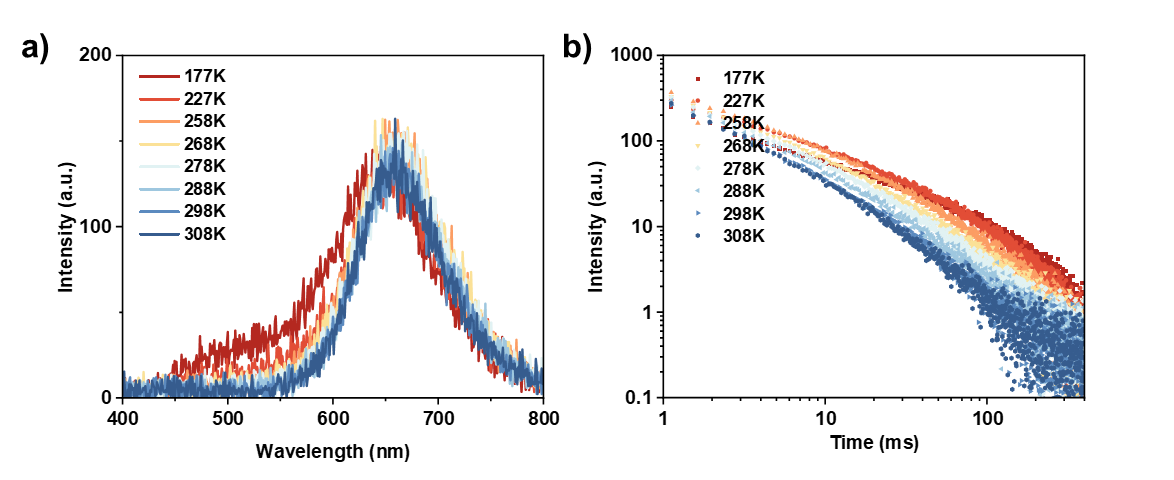


**Fig. S7.** a) Temperature-dependent delayed spectra and b) lifetime decay curves of OBDP@CN.


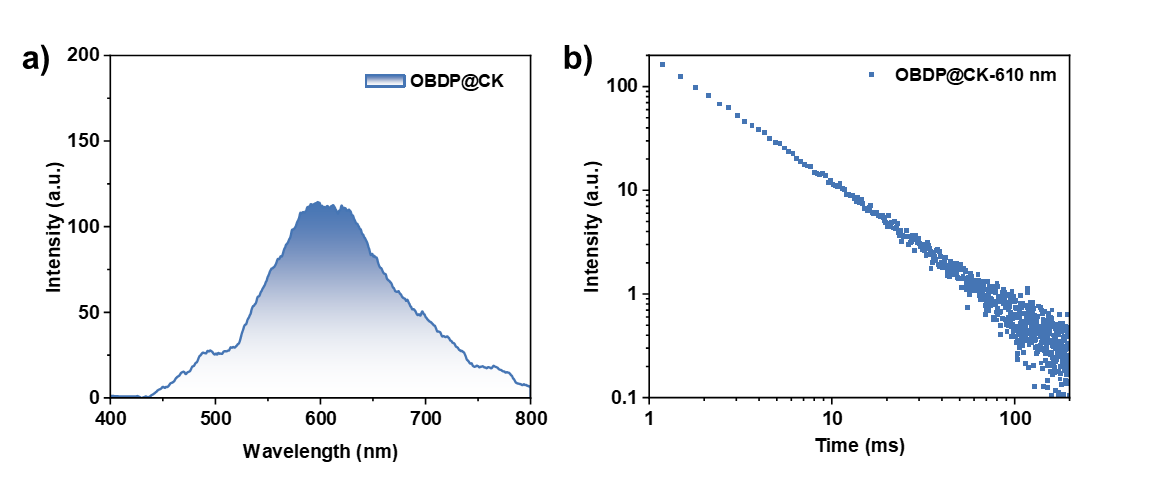


**Fig. S8.** a) The delayed spectra and b) lifetime decay curves of OBDP@CK. (doping ratio 1000: 1, λ_ex_ = 360 nm, delayed time = 0.5 ms)


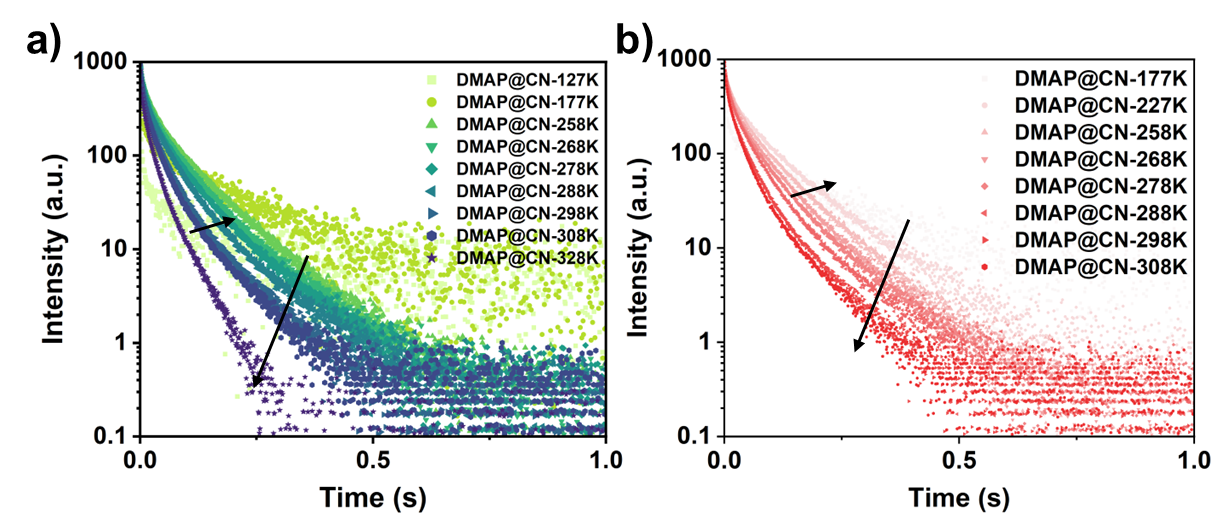


**Fig. S9.** a) and b) Temperature-dependent lifetime decay curves of BPO@CN and DMAP@CN.


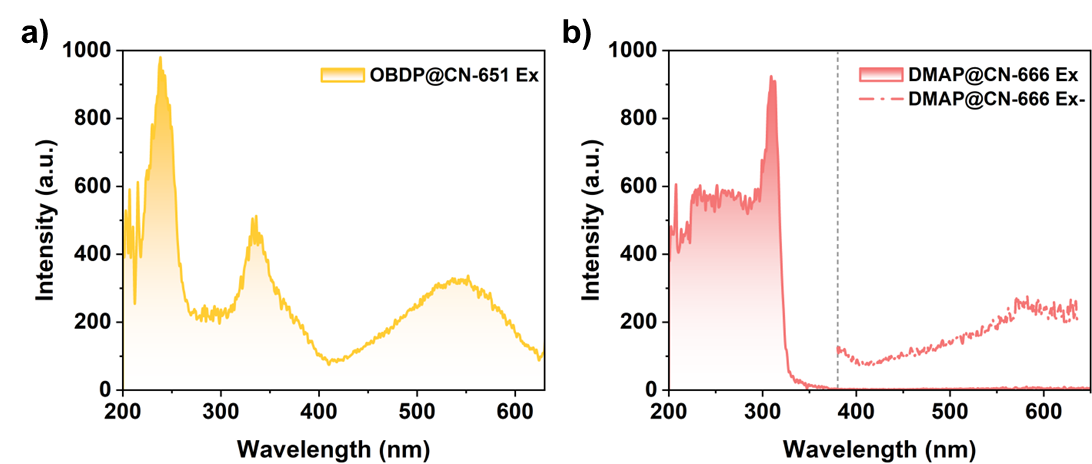


**Fig. S10.** a) and b) Excitation spectra of OBDP@CN and DMAP@CN.


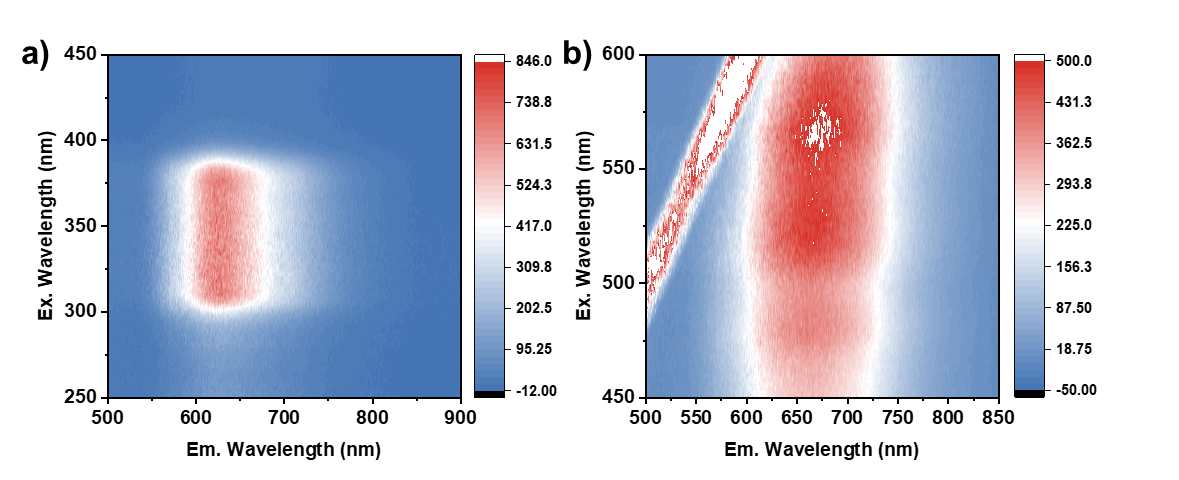


**Fig. S11.** a) and b) Emission - Excitation dependent spectra of BPO@CN.


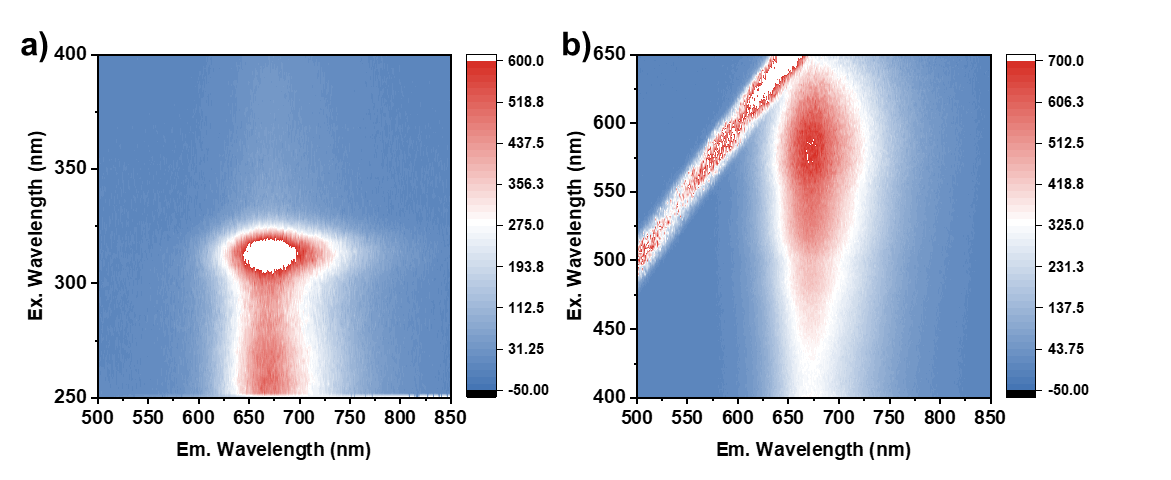


**Fig. S12.** a) and b) Emission - Excitation dependent spectra of DMAP@CN.


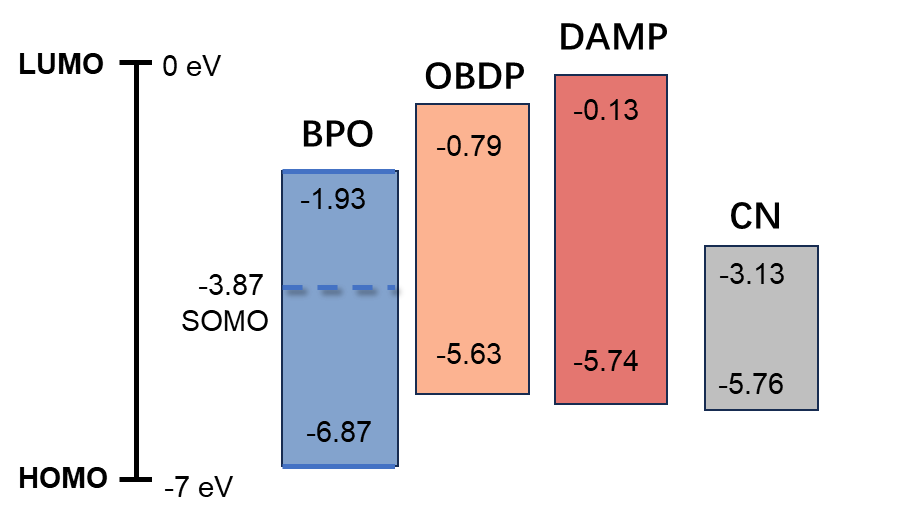


**Fig. S13.** HOMO, LUMO and SOMO distributions of BPO, OBDP, DMAP and CN.


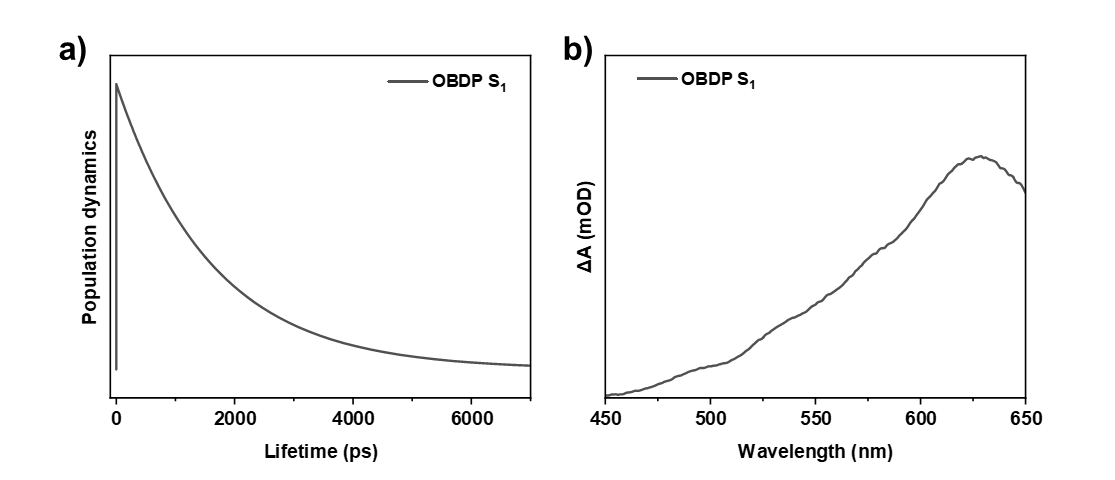


**Fig. S14.** a) and b) Population dynamics and evolution associated difference spectra of OBDP obtained through global analysis.


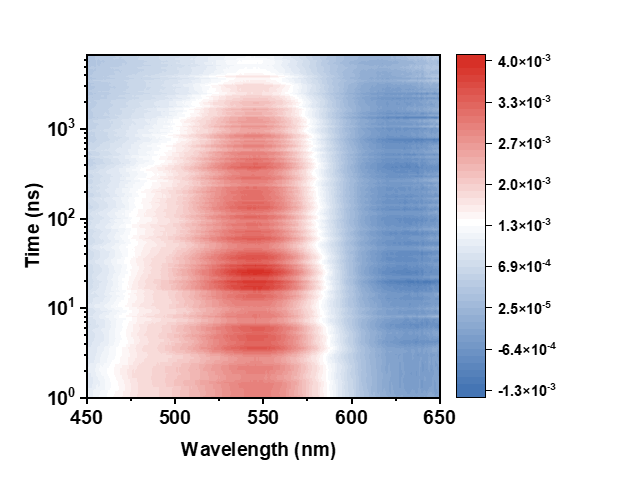


**Fig. S15.** The femtosecond TA spectra of PMMA@CN under 360 nm excitation.


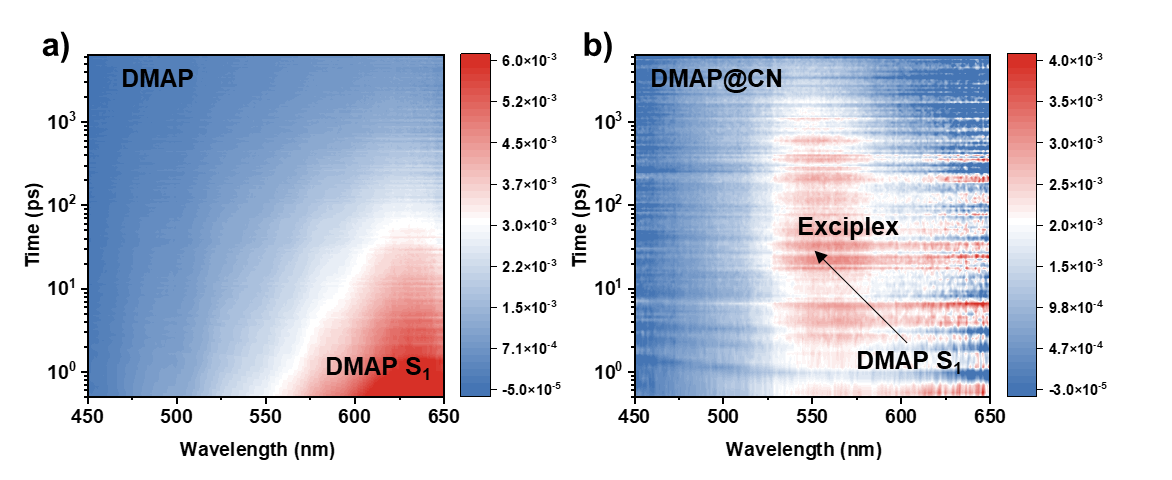


**Fig. S16.** a) and b) The femtosecond TA spectra of DMAP and DMAP@CN under 310 nm excitation.


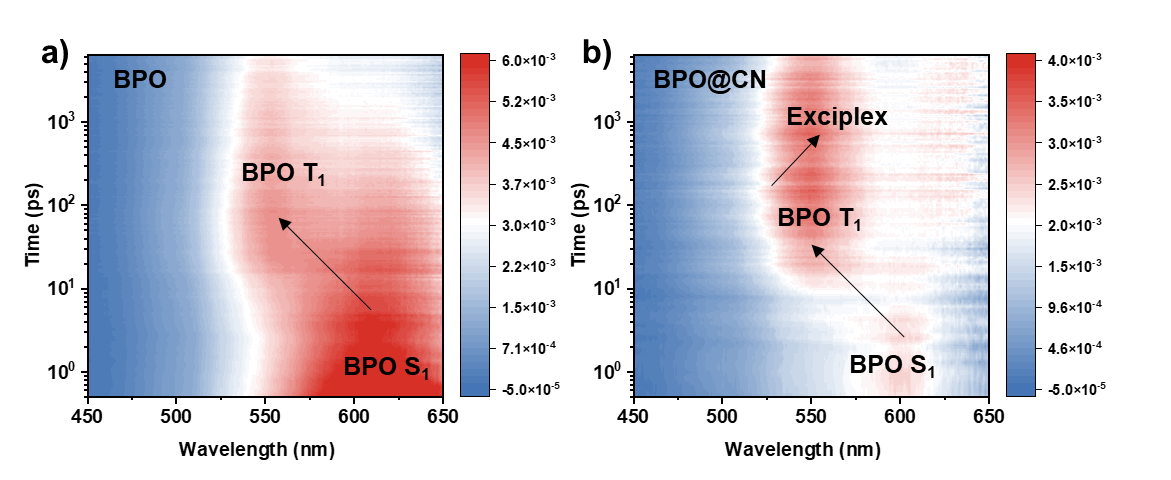


**Fig. S17.** a) and b) The femtosecond TA spectra of BPO and BPO@CN under 360 nm excitation.


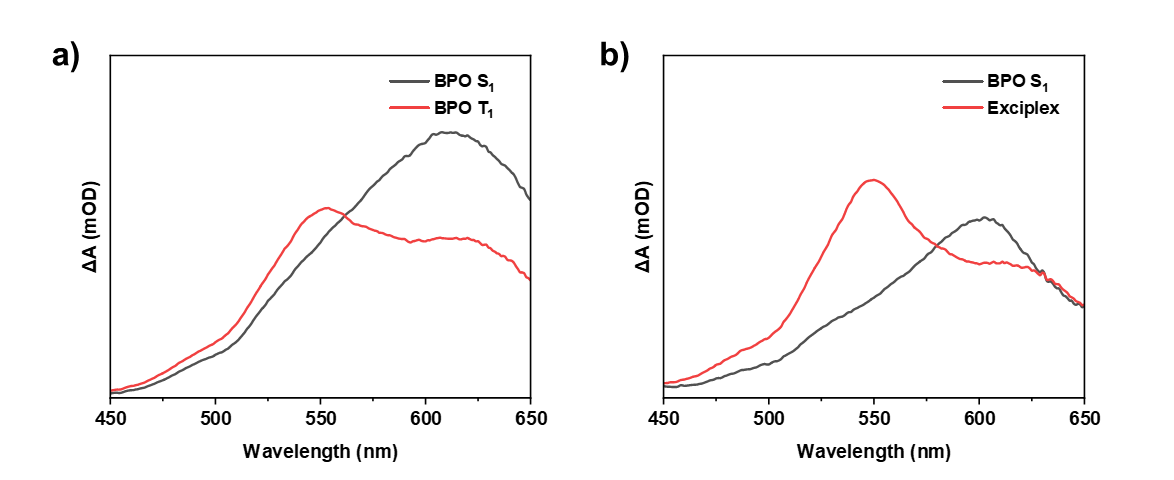


**Fig. S18.** a) and b) Evolution associated difference spectra of BPO and OBDP@CN obtained through global analysis.


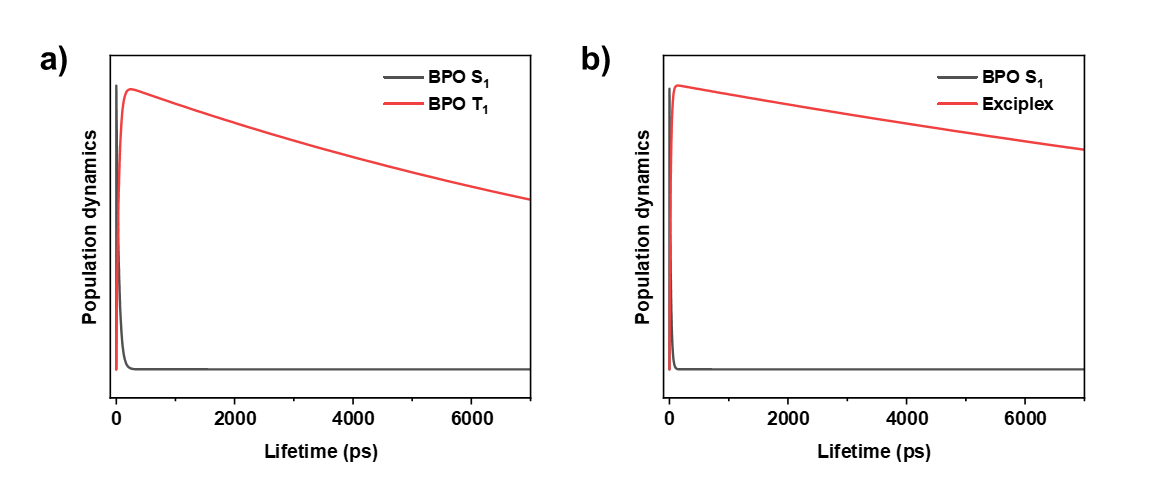


**Fig. S19.** a) and b) Population dynamics spectra of BPO and OBDP@CN obtained through global analysis.


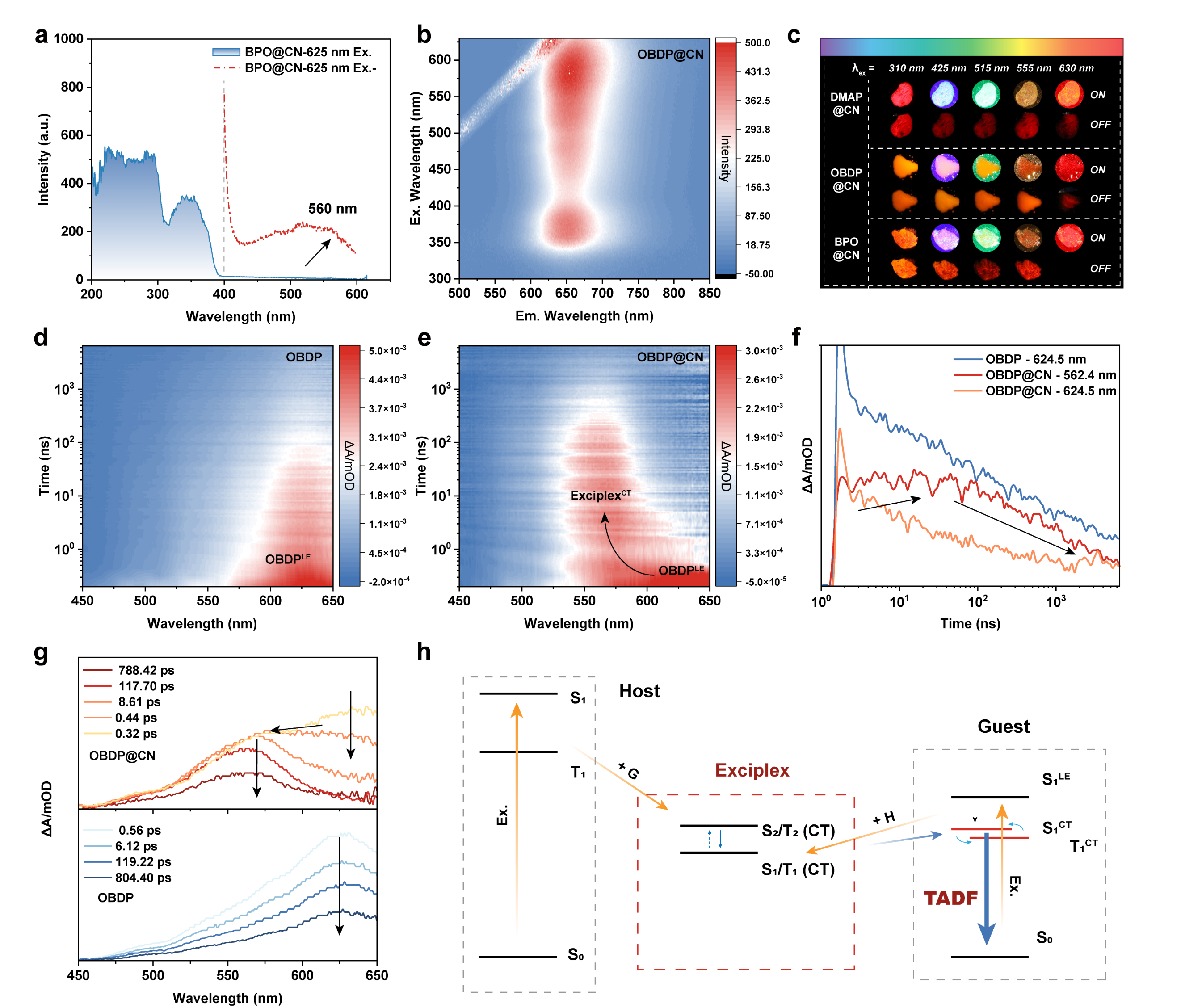


**Fig. S20.** Simplified diagram of the mechanistic process.


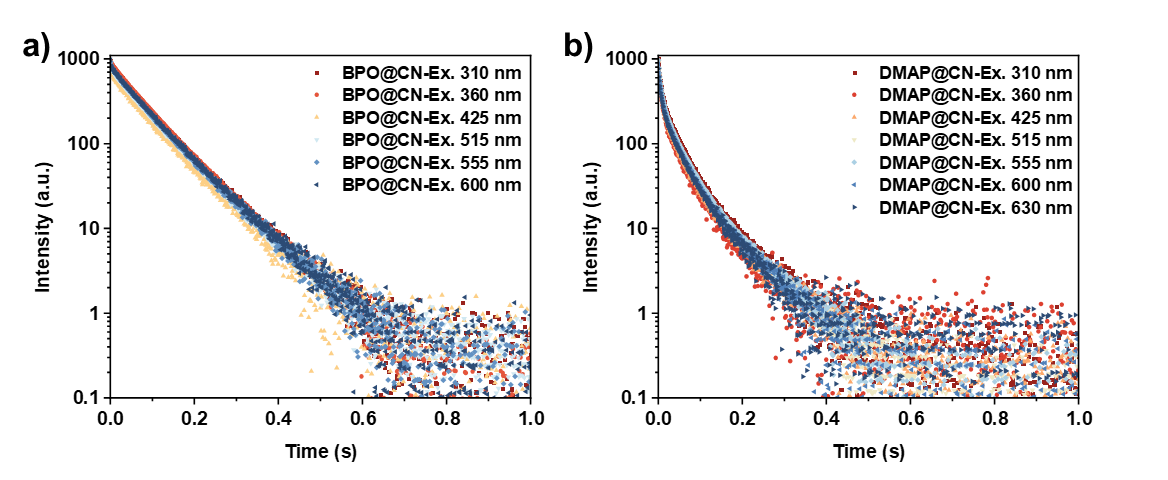


**Fig. S21.** a) and b) Lifetime decay curves of BPO@CN and DMAP@CN under different excitation wavelength.


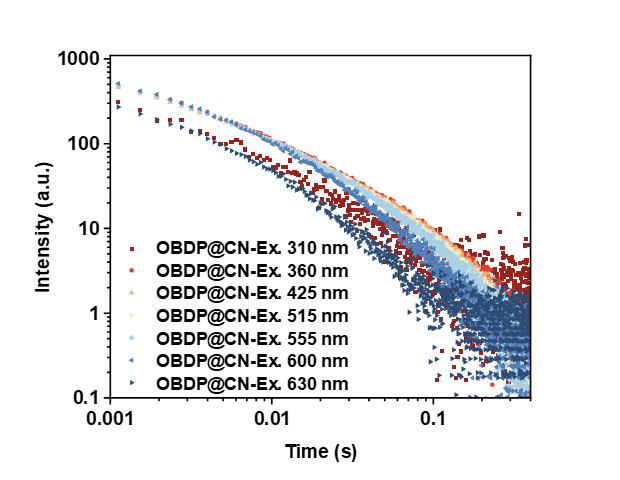


**Fig. S22.** Lifetime decay curves of OBDP@CN.

**Table S3.** PLQY of these doping materials under differeent excitation wavelength.

|  | BPO@CN | OBDP@CN | DMAP@CN |
| --- | --- | --- | --- |
| 310 nm | 23.6% | 12.1% | 18.6% |
| 360 nm | 20.7% | 71.9% | 28.0% |
| 425 nm | 16.7% | 65.3% | 22.7% |
| 515 nm | 21.1% | 70.1% | 29.1% |
| 555 nm | 18.3% | 71.1% | 29.4% |
| 600 nm | 10.5% | 68.6% | 30.5% |
| 630 nm | nd | 40.6% | 25.6% |

‘nd’ indicates not detected.


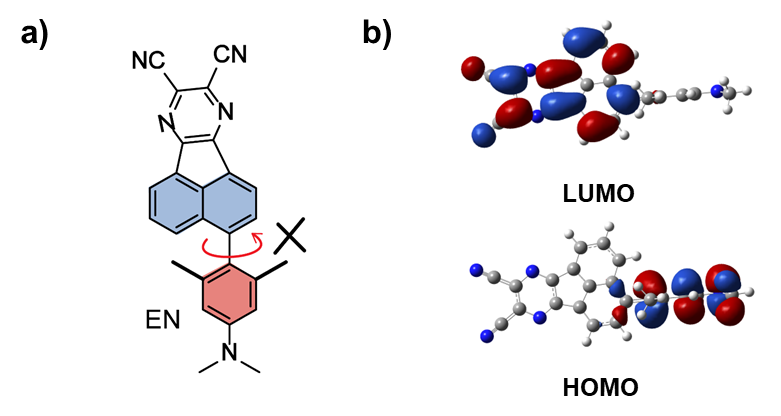


**Fig. S23.** Molecular structure (a), HOMO and LUMO distributions (b) of EN.


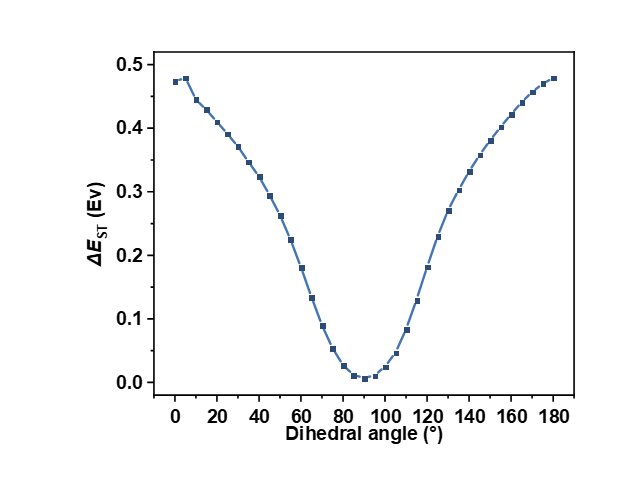


**Fig. S24.** *ΔE*_ST_ of CN with different dihedral angles.


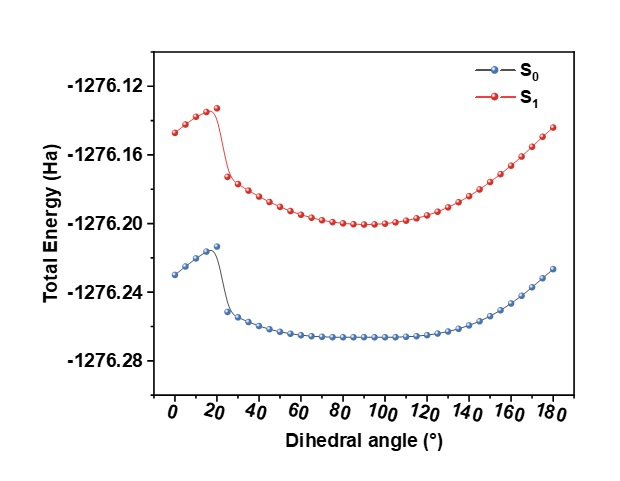


**Fig. S25.** Potential energy surface scanning of EN with different dihedral angles.


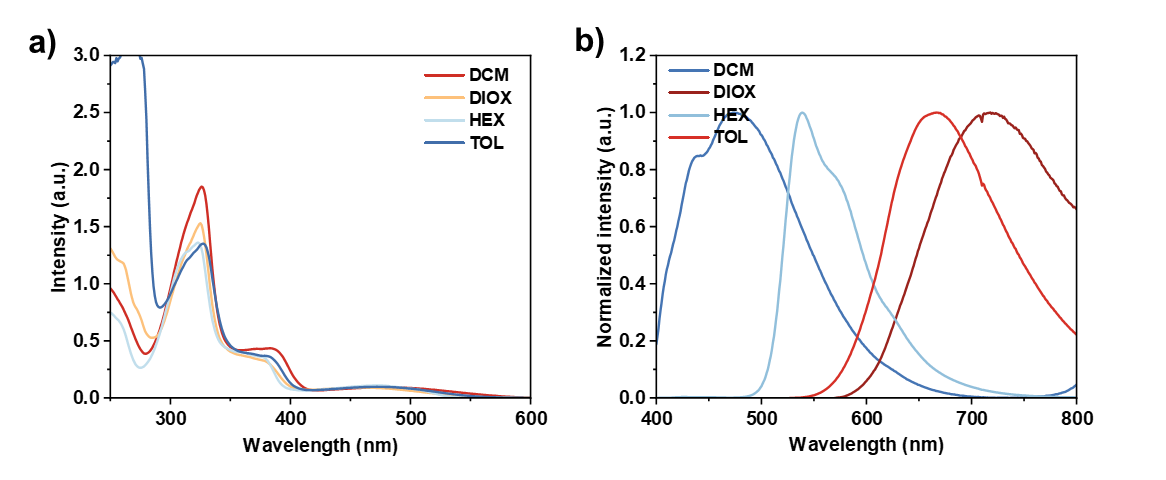


**Fig. S26.** a) Absorption and b) PL spectra of EN in different solvents. (λ_ex_ = 360 nm, C = 1×10^-5^ mol/L)


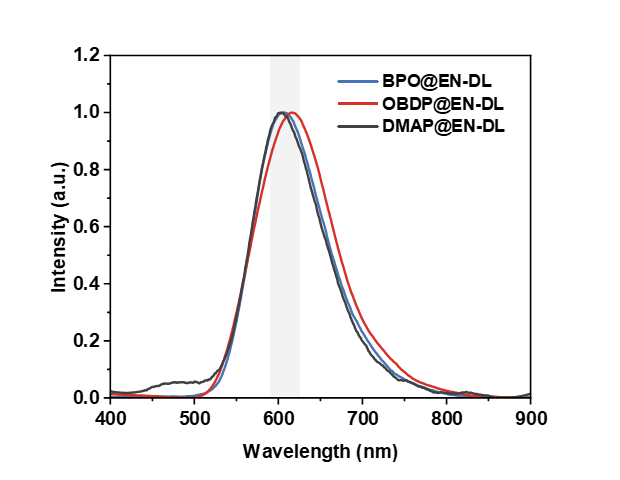


**Fig. S27.** DL spectra of BPO@EN, OBDP@EN and DMAP@EN (doping ratio 1000: 1, λ_ex_ = 360 nm, delayed time = 0.5 ms)


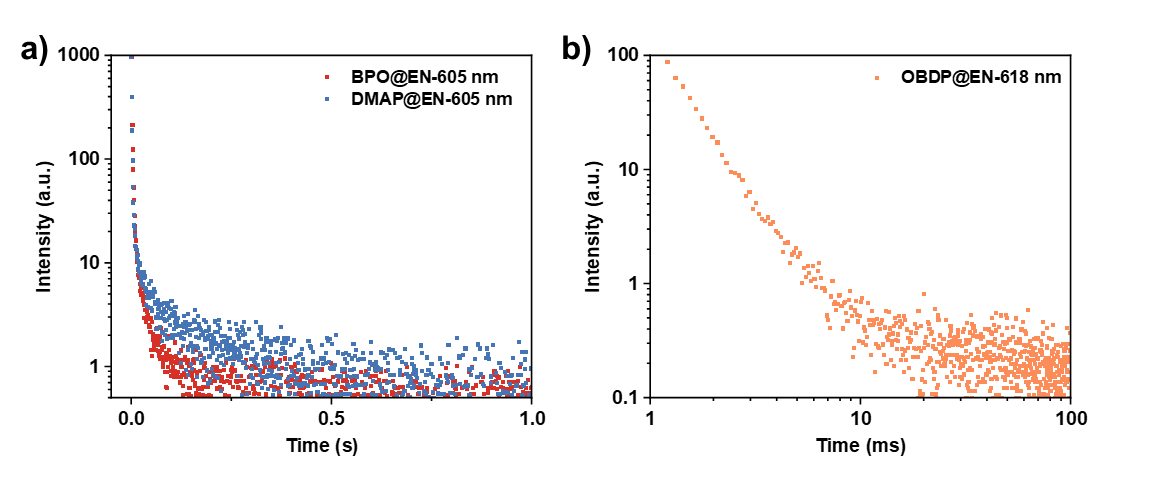


**Fig. S28.** Lifetime decay curves of a) BPO@EN, DMAP@EN and b) OBDP@EN. (doping ratio 1000: 1)


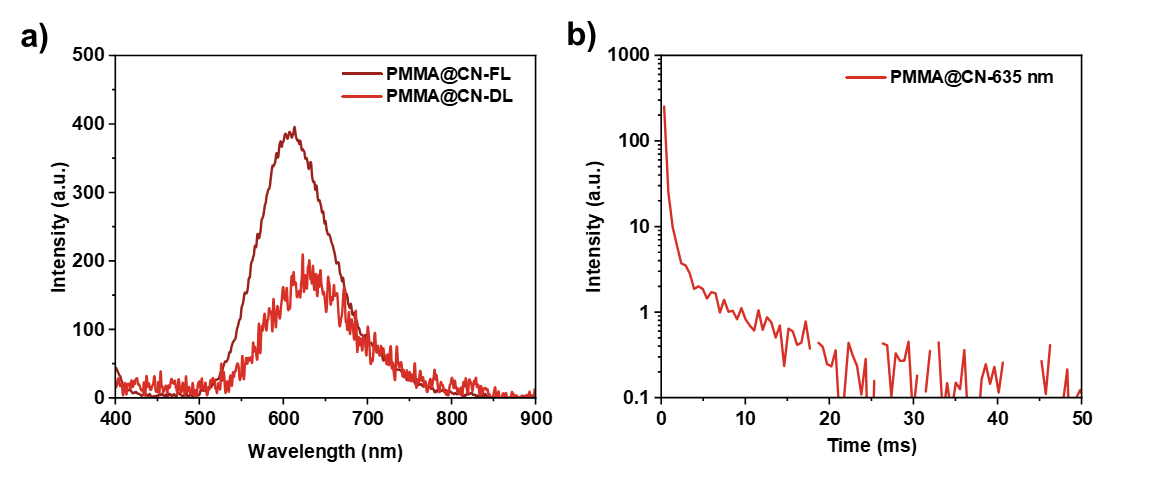


**Fig. S29.** a) Lifetime decay curves, b) the fluorescence (FL) and delayed (DL) spectra and of PMMA@CN. (doping ratio 10000: 1, λ_ex_ = 360 nm, delayed time = 0.1 ms)


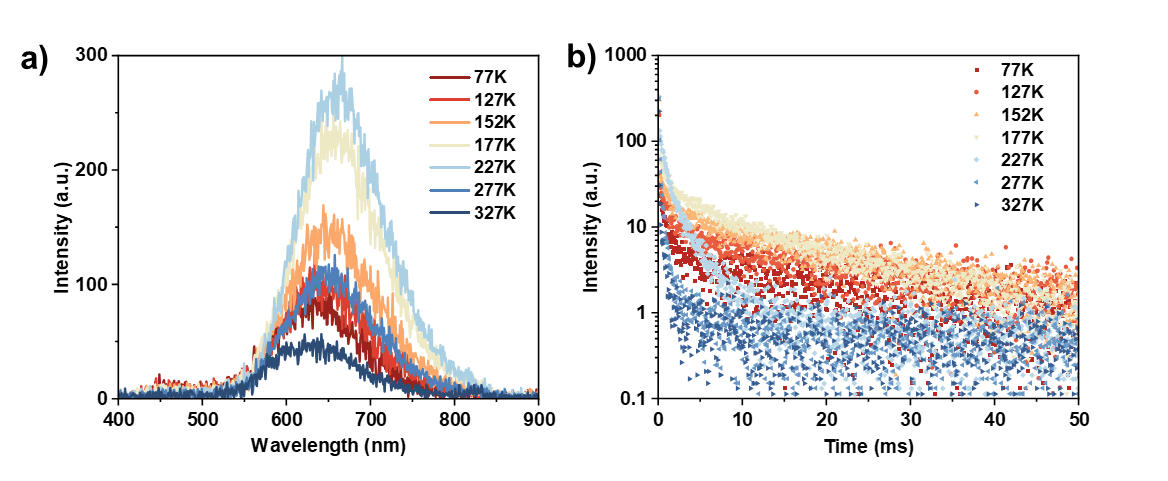


**Fig. S30.** a) Temperature-dependent delayed spectra and b) lifetime decay curves of PMMA@CN.


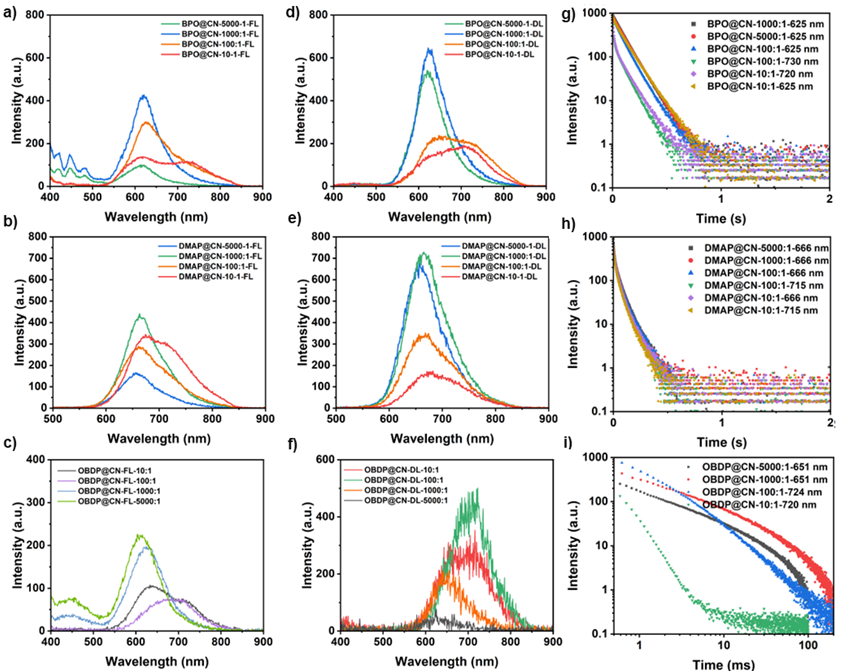


**Fig. S31.** a-d) FL, e) - h) DL emission and i) - l) lifetime decay curves of BPO@CN, DMAP@CN and OBDP@CN under different doping ratios.


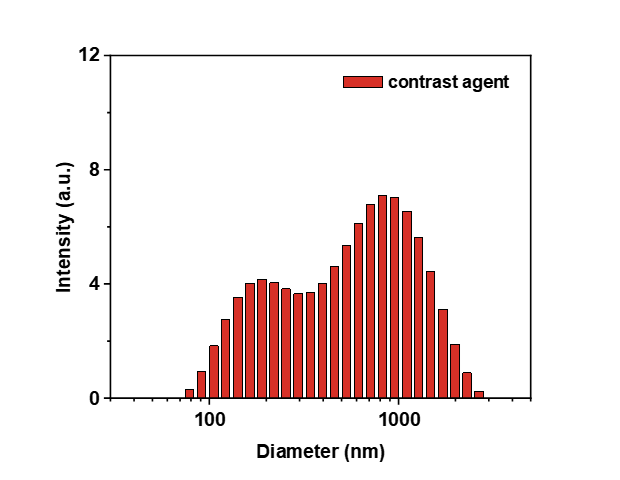


**Fig. S32.** DLS data of the contrast agent.


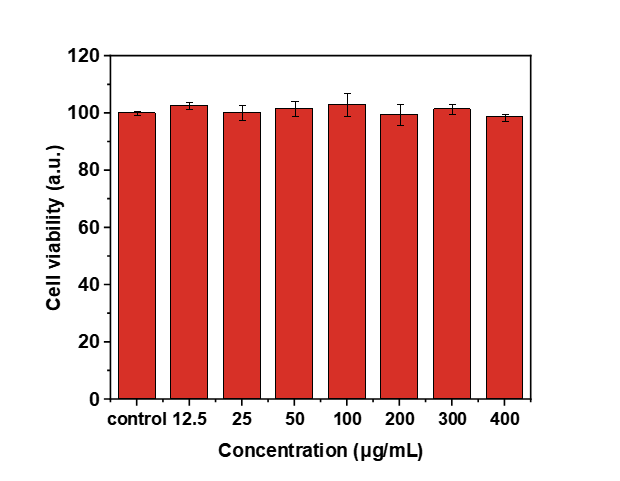


**Fig. S33.** Cell viability of HeLa cells for the contrast agent at different concentrations.


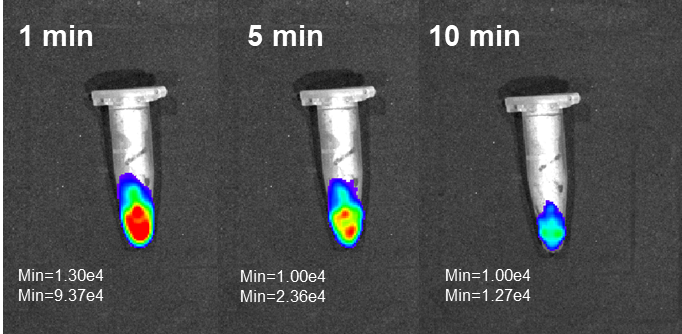


**Fig. S34.** Afterglow images of the contrast agent after 360 nm UV light irradiation.


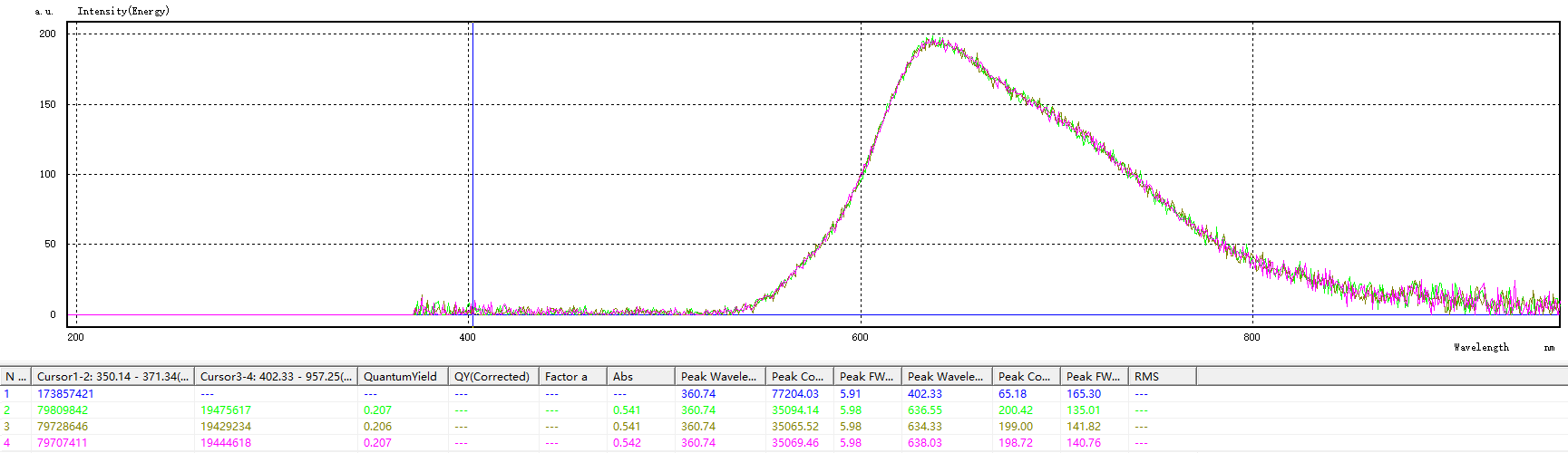


**Fig. S35.** PLQY of BPO@CN.


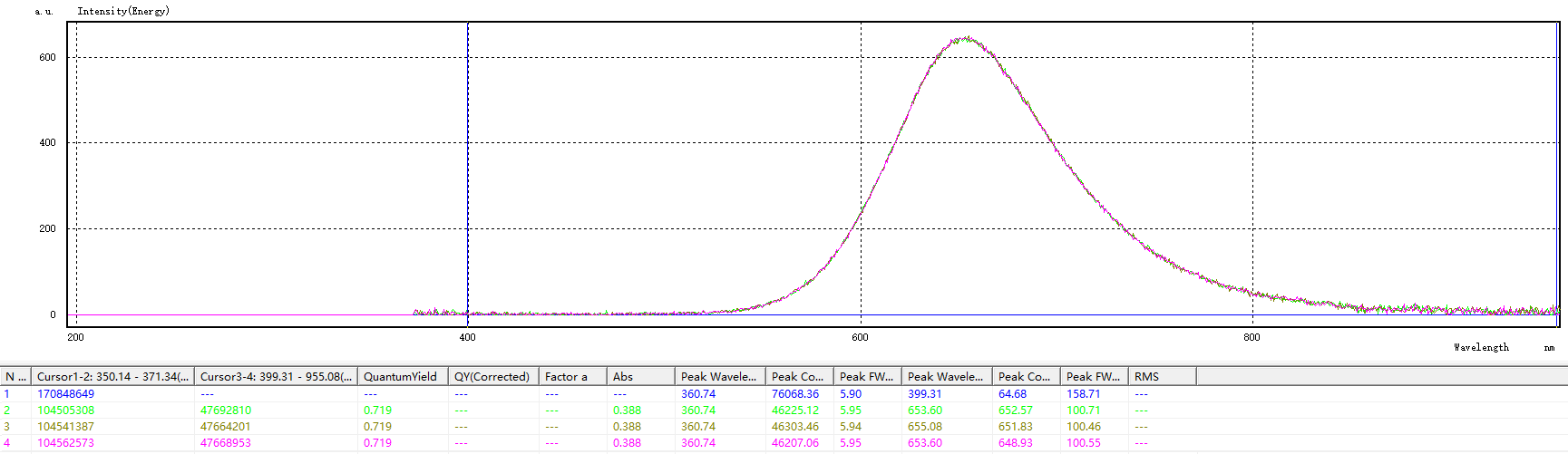


**Fig. S36.** PLQY of OBDP@CN.


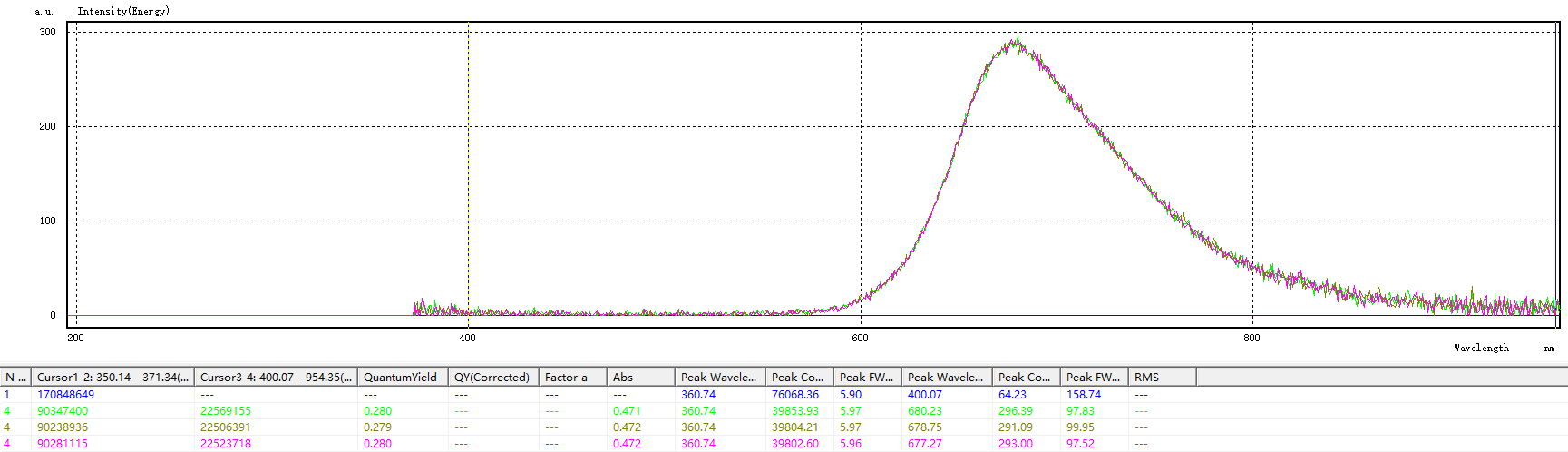


**Fig. S37.** PLQY of DMAP@CN.


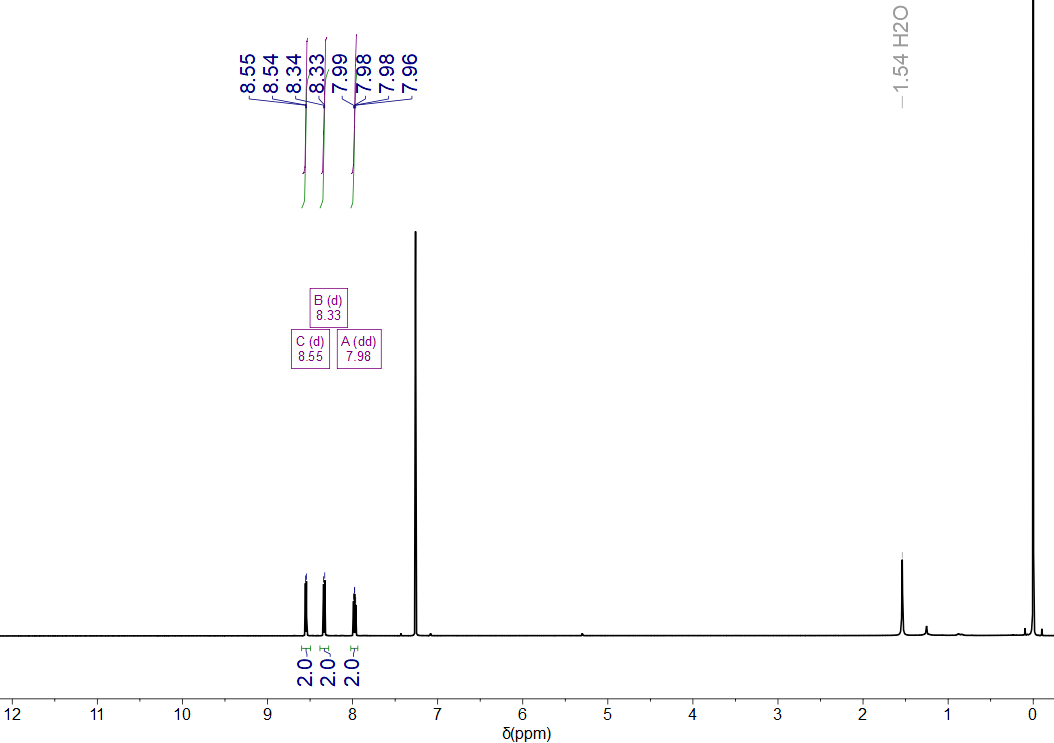


**Fig. S38.** ^1^H NMR spectrum of CK.


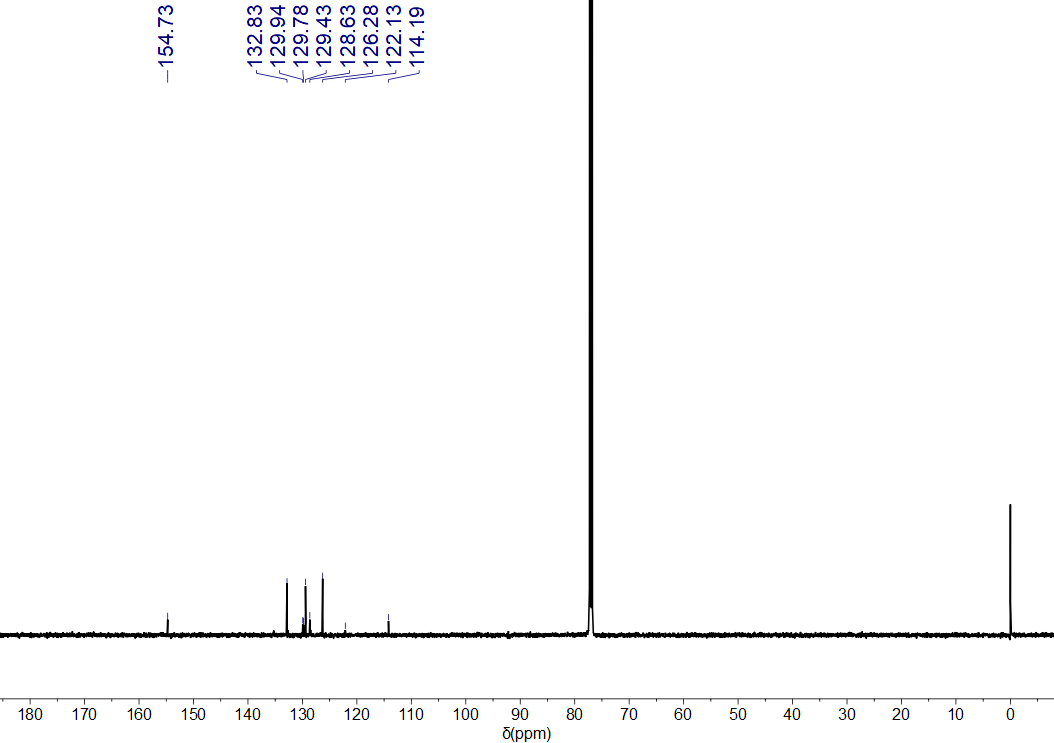


**Fig. S39.** ^13^C NMR spectrum of CK.


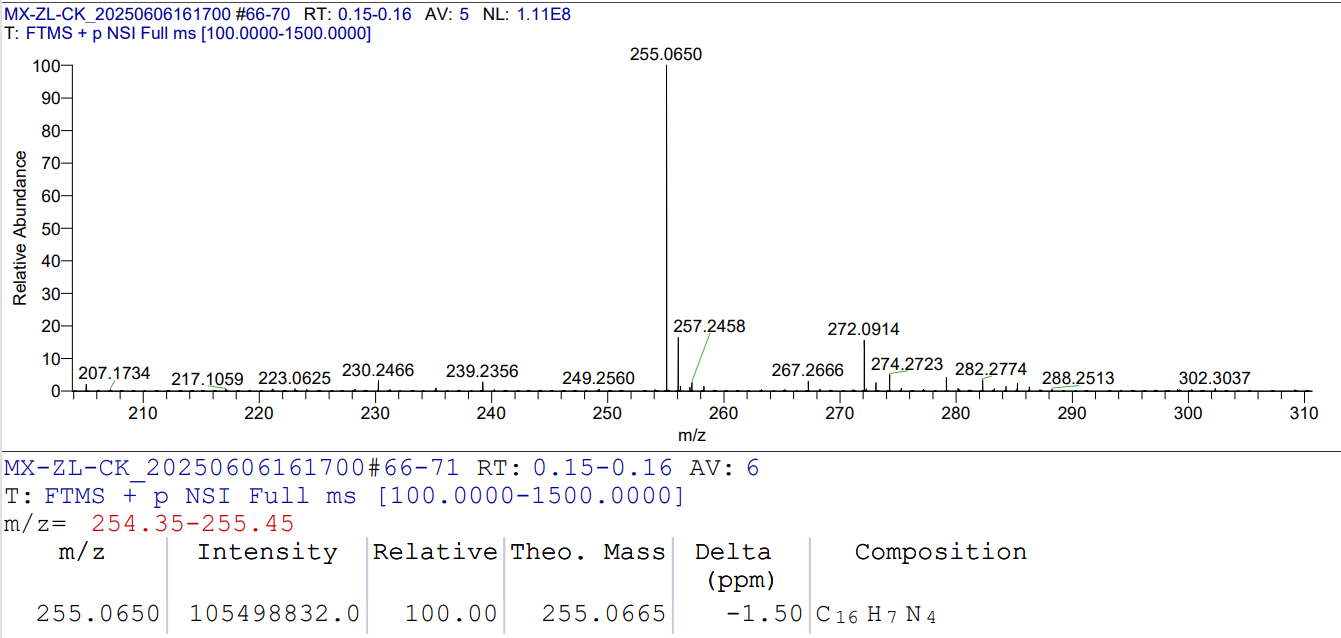


**Fig. S40.** HRMS spectrum of CK.


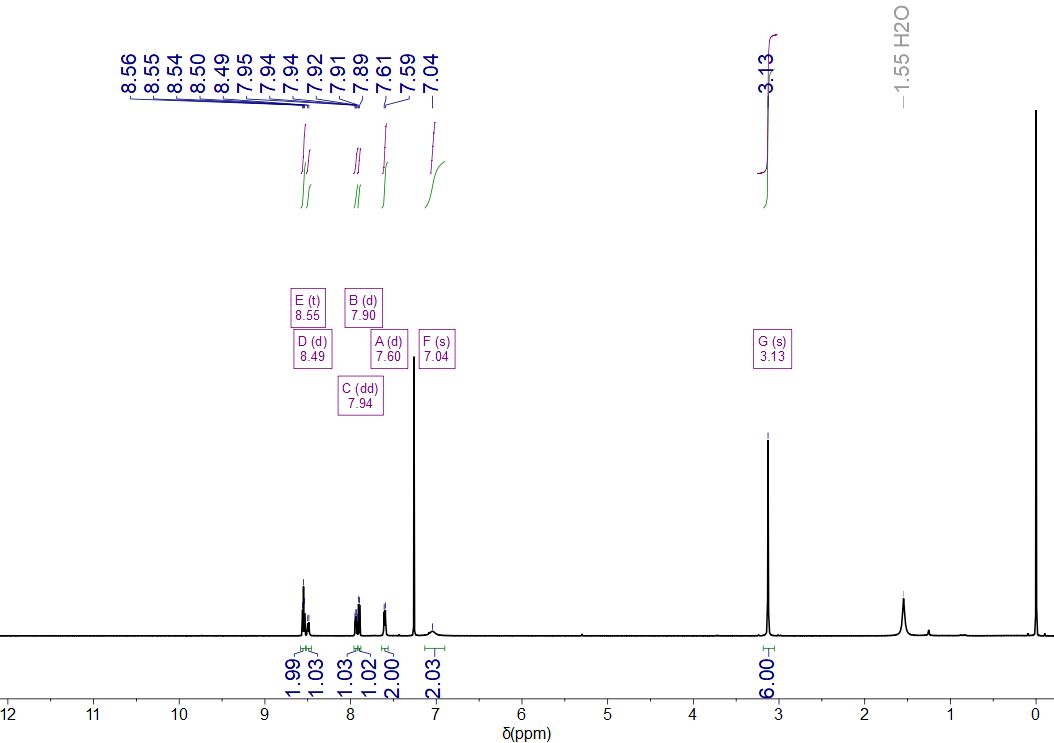


**Fig. S41.** ^1^H NMR spectrum of CN.


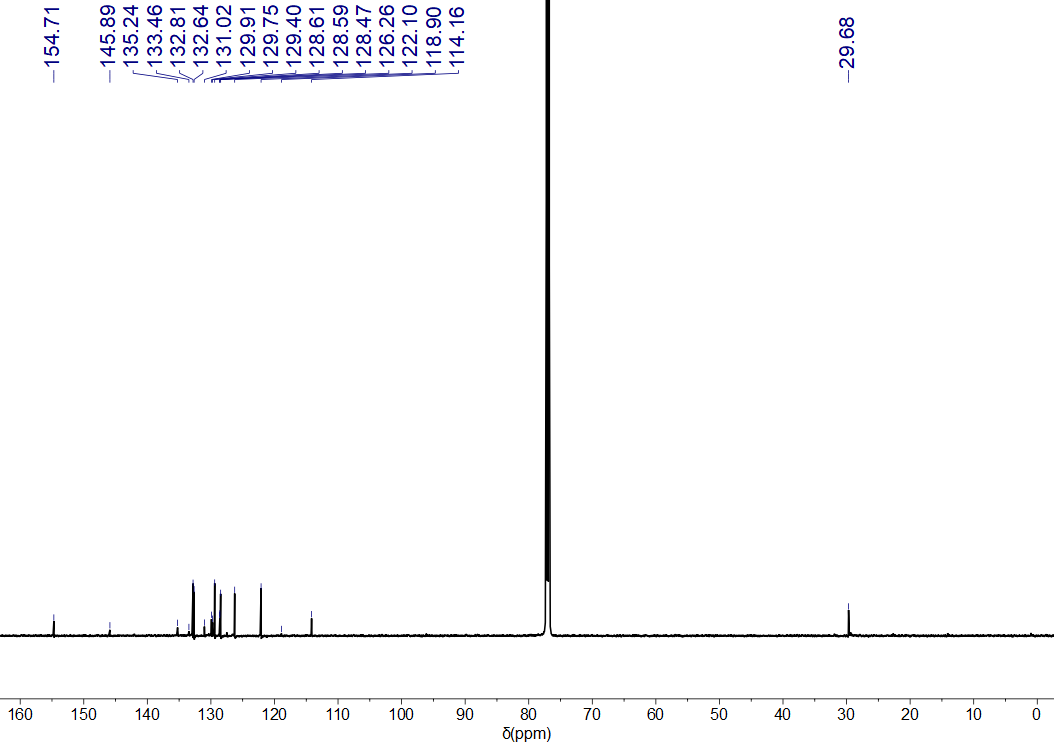


**Fig. S42.** ^13^C NMR spectrum of CN.


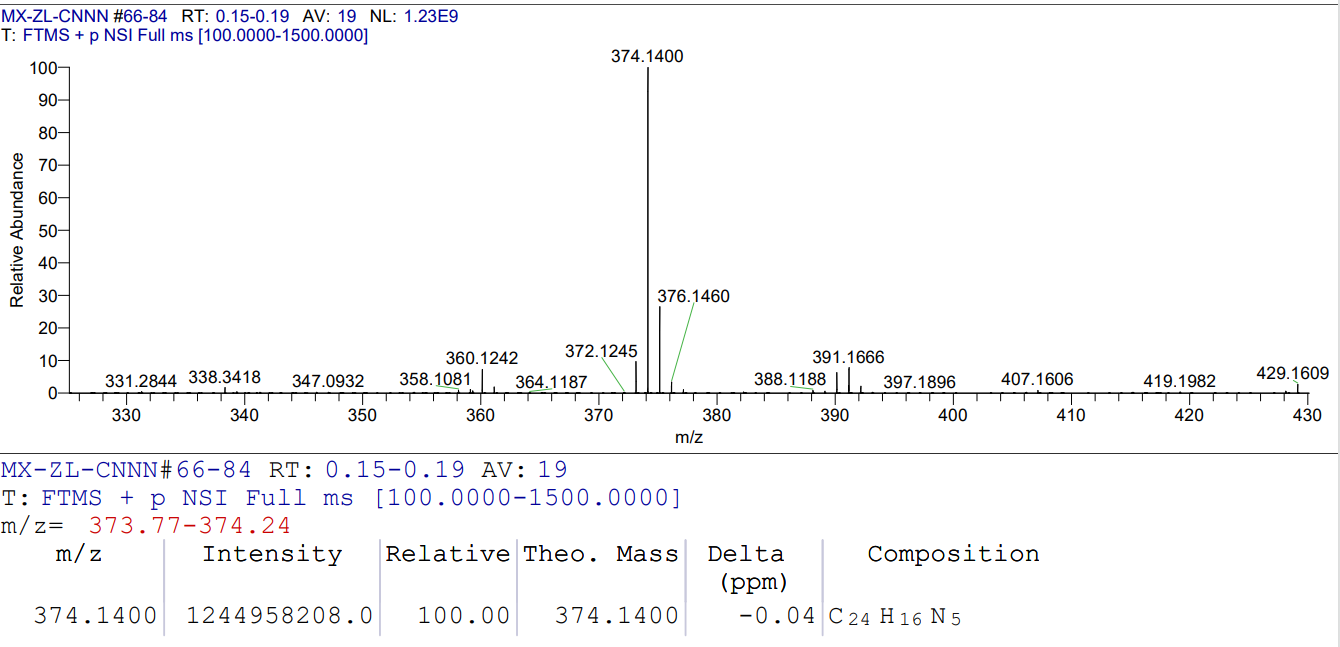


**Fig. S43.** HRMS spectrum of CN.


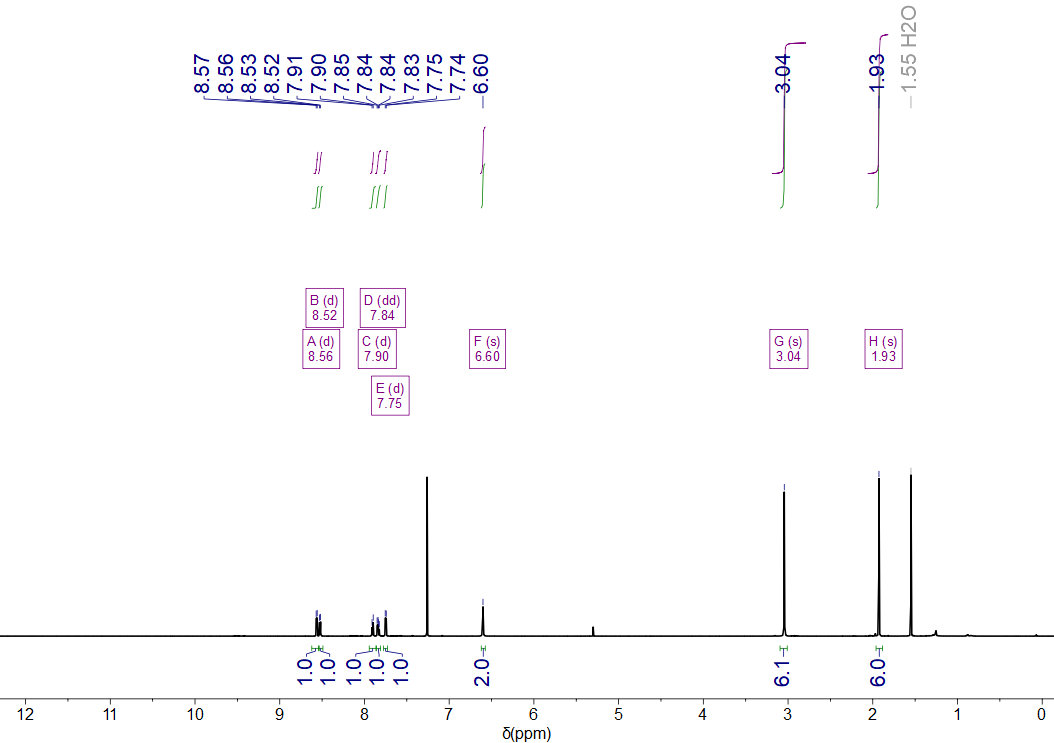


**Fig. S44.** ^1^H NMR spectrum of EN.


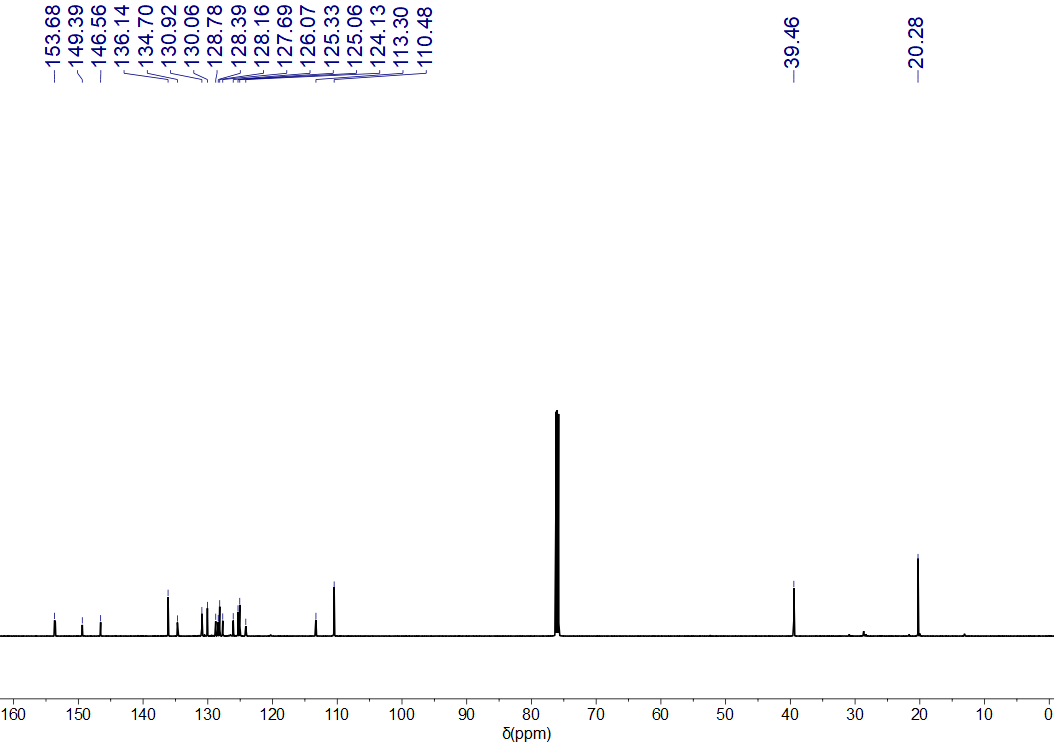


**Fig. S45.** ^13^C NMR spectrum of EN.


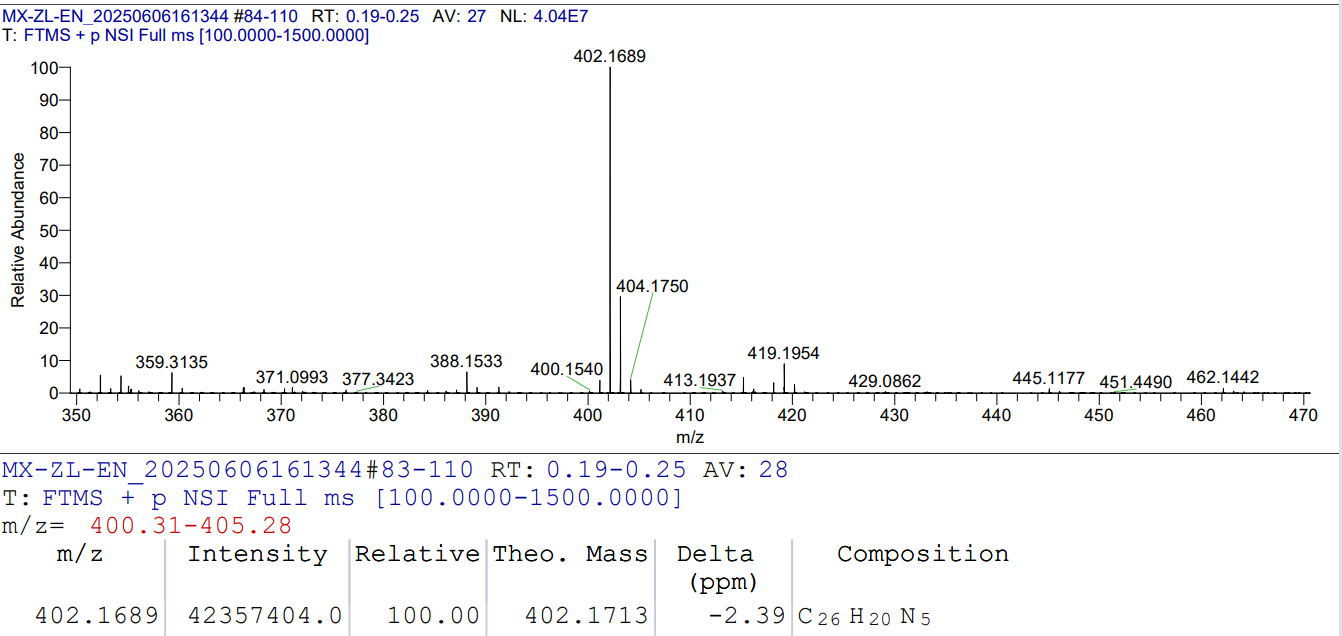


**Fig. S46.** HRMS spectrum of EN.

**References**

1. Kalhor, M., Shayestefar, M., Khalaj, M. & Janghorban, F. Ca(IO3)2 nanoparticles: fabrication and application as an eco-friendly and recyclable catalyst for the green synthesis of quinoxalines, pyridopyrazines, and 2,3-dicyano pyrazines. *Research on Chemical Intermediates* **49**, 885–900 (2022).
2. Bao, X. et al. Dual-mode thermochromic afterglow in phosphorus-doped carbon dot composites for visible light-activated information encryption. *Journal of Colloid and Interface Science* **690**, 137331 (2025).
3. Liu, J. et al. Coronene doped rubber-toughened plastics: easily photoactivatable, visible light excitable, stress-whitening quenching and thermally recoverable ultralong phosphorescence. *ACS Materials Letters* **7**, 876–883 (2025).
4. Chen, W.-G. et al. Visible-light excitable polymers with ultra-long phosphorescence afterglow achieved by boron-based dative bonds. *Chemical Engineering Journal* **503**, (2025).
5. Du, B. et al. Multi-resonance emitters with room-temperature phosphorescence in amorphous state and excited by visible light. *Chemical Science* **15**, 19432–19442 (2024).
6. You, J. et al. Responsive circularly polarized ultralong room temperature phosphorescence materials with easy-to-scale and chiral-sensing performance. *Nature Communications* **15**, 7149 (2024).
7. Zheng, Y., Li, Z. & Zhang, H. Visible-excitable long-afterglow material with dual-mode emission of delayed fluorescence and room temperature phosphorescence. *Journal of Materials Chemistry C* **12**, 11506–11512 (2024).
8. Guo, D. et al. Visible-light-excited robust room-temperature phosphorescence of dimeric single-component luminophores in the amorphous state. *Nature Communications* **15**, 3598 (2024).
9. Wang, X. et al. Multicolor ultralong organic phosphorescence through alkyl engineering for 4d coding applications. *Chemistry of Materials* **31**, 5584–5591 (2019).
10. Wang, X.-F. et al. Pure organic room temperature phosphorescence from excited dimers in self-assembled nanoparticles under visible and near-infrared irradiation in water. *Journal of the American Chemical Society* **141**, 5045–5050 (2019).
11. Zhang, L. et al. White light-excited organic room-temperature phosphorescence for improved in vivo bioimaging. *Nature Communications* **16**, 3970 (2025).
12. Fan, Y. et al. Mobile phone flashlight‐excited red afterglow bioimaging. *Advanced Materials* **34**, 2201280 (2022).
13. Garain, S. et al. Arylene diimide phosphors: aggregation modulated twin room temperature phosphorescence from pyromellitic diimides. *Angewandte Chemie International Edition* **60**, 12323–12327 (2021).
14. Zheng, Y. et al. Long-lived room temperature phosphorescence crystals with green light excitation. *ACS Applied Materials & Interfaces* **14**, 15706–15715 (2022).
15. Wang, X. F. et al. Pure organic room temperature phosphorescence from unique micelle‐assisted assembly of nanocrystals in water. *Advanced Functional Materials* **30**, 1907282 (2020).
16. Liang, Y. et al. Color‐tunable dual‐mode organic afterglow from classical aggregation‐caused quenching compounds for white‐light‐manipulated anti‐counterfeiting. *Angewandte Chemie International Edition* **62**, e202217616 (2023).
17. Cai, S., Shi, H., Li, J., Gu, L., Ni, Y., Cheng, Z. et al. Visible‐light‐excited ultralong organic phosphorescence by manipulating intermolecular interactions. *Advanced Materials* **29**, 1701244 (2017).
18. Jin, J. et al. Modulating tri‐mode emission for single‐component white organic afterglow. *Angewandte Chemie International Edition* **60**, 24984–24990 (2021).
19. Nie, X. et al. Broad‐band visible‐light excitable room‐temperature phosphorescence via polymer site‐isolated dye aggregates. *Advanced Optical Materials* **10**, 2200099 (2022).
20. Lin, Z., Li, M., Yoshioka, R., Oyama, R. & Kabe, R. Oxygen-tolerant near-infrared organic long-persistent luminescent copolymers. *Angewandte Chemie International Edition* **63**, e202314500 (2024).
